# Solvent effects on the radical scavenging activity of HP-136 benzofuranone: mechanistic and kinetic insights from quantum chemical calculations

**DOI:** 10.1039/d6ra04239b

**Published:** 2026-07-15

**Authors:** Tran Duc Manh, Vo Thao My, Vo Huynh Ngoc Diep, Truong Le Bich Tram, Mai Van Bay, Tran Thanh Diep, Nguyen Quang Trung, Adam Mechler, Quan V. Vo

**Affiliations:** a The University of Danang – University of Sciences and Education Danang 550000 Vietnam; b The University of Danang – University of Technology and Education Danang 550000 Vietnam vvquan@ute.udn.vn; c Department of Science and International Cooperation, The University of Danang Danang 550000 Vietnam; d Center of National Defense – Security Education and Sports, The University of Danang Vietnam; e Quality Assurance and Testing Center 2 Da Nang 550000 Vietnam; f Department of Biochemistry and Chemistry, La Trobe University Victoria 3086 Australia

## Abstract

The radical-scavenging activity of Irganox HP-136 (5,7-di-*tert*-butyl-3-(3,4-dimethylphenyl)-3*H*-benzofuran-2-one), a representative benzofuranone antioxidant used in polymer stabilization, was systematically investigated using quantum chemical calculations. The reactivity of HP-136 toward a series of environmentally relevant radicals was evaluated, with particular emphasis on the highly reactive hydroxyl radical (HO˙). In the gas phase, HP-136 exhibited outstanding HO˙ scavenging activity, with an overall rate constant of 2.06 × 10^10^ M^−1^ s^−1^, predominantly governed by formal hydrogen transfer (FHT) at the C3–H and C12–H positions. In contrast, reactions with HOO˙ radicals were several orders of magnitude slower. In benzene, HP-136 showed high reactivity toward the *tert*-butoxyl radical (TBO˙), with a calculated rate constant of 3.50 × 10^5^ M^−1^ s^−1^, in good agreement with the experimental value (1.35 × 10^5^ M^−1^ s^−1^). HP-136 exhibited relatively low scavenging activity toward HOO˙ and 2-cyanoprop-2-yl (AI˙) radicals in both lipid and polar media. The reactivity toward TBO˙ and cumyloxyl (CMO˙) radicals was moderate in benzene and DMSO, with overall rate constants ranging from 10^3^ to 10^5^ M^−1^ s^−1^. The results further revealed that the sequential proton loss-electron transfer (SPLET) mechanism is not rate-determining in the DPPH˙, and AI˙ scavenging reactions of HP-136 in DMSO, although it contributes significantly to the antiradical activity toward TBO˙ and CMO˙ radicals in polar aprotic media.

## Abstract 2

Irganox HP-136 exhibits medium-to-high, solvent-dependent radical-trapping capacity with tunable mechanism.

## Graphical abstract

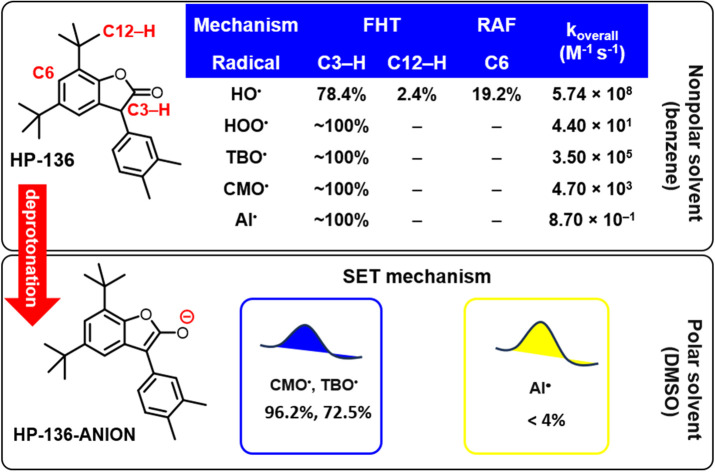

## 1. Introduction

**Fig. 1:**
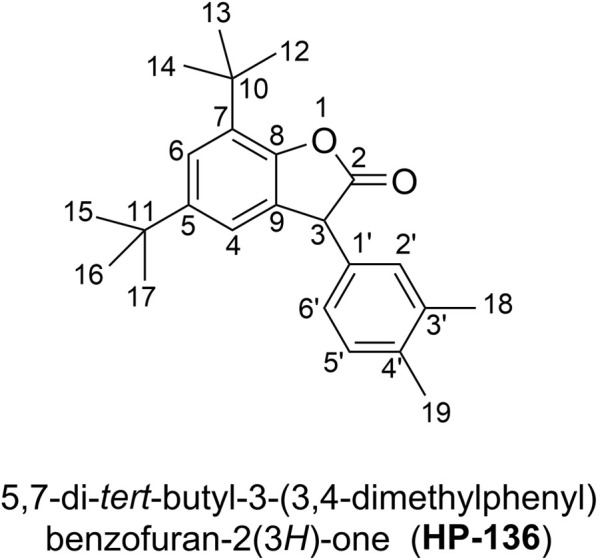
Structure of HP-136.

Polymeric materials inevitably undergo chemical and physical changes when subjected to various thermal and oxidative environments during processing and long-term use. Recent studies have emphasized that such degradation phenomena – including chain scission, crosslinking, discoloration, and the gradual loss of mechanical integrity – are primarily driven by complex radical-mediated oxidation pathways involving alkyl, peroxyl, and alkoxyl species.^1–4^ The suppression of these radical chain reactions therefore represents a central strategy in polymer stabilization, commonly achieved through the incorporation of antioxidant additives. While hindered phenols remain the most widely used stabilizers, increasing attention has been directed toward benzofuranone-type antioxidants, which constitute a distinct group of chain-breaking agents noted for their effectiveness under high-temperature processing conditions and their complementary behavior in advanced stabilization systems.^2,3^ Among them, Irganox HP-136 (Fig. 1) represents a typical and commercially important benzofuranone antioxidant. HP-136 is composed mainly of 5,7-di-*tert*-butyl-3-(3,4-dimethylphenyl)-3*H*-benzofuran-2-one, accompanied by a minor amount of its positional isomer, 5,7-di-*tert*-butyl-3-(2,3-dimethylphenyl)-3*H*-benzofuran-2-one. Previous studies have demonstrated that HP-136 behaves as a medium-strength chain-breaking antioxidant during polypropylene oxidation at elevated temperatures (180–200 °C). Moreover, its stabilizing efficiency can be significantly enhanced in the presence of phosphites, sulphides, or phenolic antioxidants, indicating a pronounced synergistic effect within multicomponent stabilization systems.^5^

From a structural and mechanistic perspective, HP-136 belongs to the broader family of 3-aryl-benzofuran-2-one antioxidants, whose chemical behavior is closely related to their tautomeric and electronic structures. Systematic investigations involving HP-136 and numerous benzofuranone analogues have revealed that the lactone form is thermodynamically favored, both in the solid state and in solution, whereas the enol form does not constitute a noticeable population under typical conditions. This structural preference has important implications for the antioxidant activity of benzofuranones, as the lactone framework facilitates the formation of relatively stable radical intermediates during radical scavenging processes.^6^ Beyond thermal stabilization, HP-136 has also been shown to exhibit remarkable UV stabilization performance. Comparative spectroscopic studies using infrared and UV-visible techniques, combined with non-isothermal chemiluminescence measurements, demonstrated that although HP-136 is a relatively weak thermo-oxidative stabilizer, it acts as an efficient UV stabilizer for polypropylene films. This behavior has been attributed to a photochemical transformation of HP-136 into 2-hydroxybenzophenone-type moieties, which are capable of absorbing ultraviolet radiation while simultaneously scavenging reactive free radicals.^7^ At the kinetic level, the radical-trapping ability of benzofuranone antioxidants has been explored mainly through model reactions involving alkoxyl radicals. Laser flash photolysis studies on several 3-aryl-benzofuranones have provided quantitative rate constants for hydrogen donation toward *tert*-butoxyl radicals, revealing a strong dependence of antioxidant activity on substituent position and molecular structure. In particular, the presence of a methyl group at the *ortho* position of the aryl ring was found to hinder hydrogen transfer, resulting in reduced radical-scavenging efficiency.^8^ The results suggest that slight differences in structure can lead to noticeable changes in the behavior of benzofuranone antioxidants.

Despite these advances, previous studies on HP-136 and related benzofuranone antioxidants have mainly focused on polymer stabilization performance, UV stabilization behavior, or selected hydrogen-transfer reactions involving specific radical species. Experimental studies reported that HP-136 reacts with *tert*-butoxyl radical (TBO˙) in benzene with a rate constant of 1.35 × 10^5^ M^−1^ s^−1^,^9^ while related benzofuranone antioxidants generally exhibit radical-scavenging reactivity in the range of 10^5^–10^7^ M^−1^ s^−1^ in organic media.^10–12^ Zhu *et al.* further suggested that under strongly polar aprotic conditions such as DMSO, deprotonated forms may play an important role in determining the antioxidant behavior of benzofuranone systems.^6^ However, a systematic kinetic investigation of the competing formal hydrogen transfer (FHT), radical adduct formation (RAF), and single electron transfer (SET) pathways for HP-136 itself across different solvent environments remains lacking. In particular, solvent-dependent mechanistic competition between these pathways under nonpolar and polar aprotic conditions has not yet been comprehensively clarified. Elucidating these effects is important for establishing structure–reactivity relationships in benzofuranone antioxidants and for improving mechanistic understanding of their radical-scavenging behavior under oxidative conditions.

In recent years, computational (*in silico*) approaches have gained widespread acceptance as powerful tools for elucidating the mechanisms and kinetics of radical-mediated processes. Advanced quantum chemical methodologies enable the generation of reliable thermodynamic and kinetic parameters with substantially reduced experimental cost and time investment.^13–15^ In the present work, a well-established quantum chemistry-based protocol,^6,19^ is employed to systematically investigate the interactions between HP-136 and representative radical species, including HO˙, HOO˙, *tert*-butoxyl (TBO˙), cumyloxyl (CMO˙), and 2-cyanoprop-2-yl (AI˙) radicals, in typical organic solvent environments, namely benzene and dimethyl sulfoxide.

## 2. Computational details

The reaction between HP-136 and the HO˙ can proceed through several fundamental pathways, including formal hydrogen transfer (FHT), radical adduct formation (RAF), and single-electron transfer (SET), as illustrated in schemes (1)–(3).^13,14,16–18^

1 FHT: HP-136-H + HO˙ → HP-136˙ + H_2_O

2 SET: HP-136-H + HO˙ → [HP-136-H]˙^+^ + HO^−^

3 RAF: HP-136-H + HO˙ → [HO-HP-136-H]˙

The kinetic parameters were determined using the quantum mechanics-based test for overall free radical scavenging activity (QM-ORSA) methodology,^19^ which is particularly suitable for evaluating radical-mediated reactions.^13,14,20,21^ The calculations were conducted within the framework of transition state theory (TST) at 298.15 K, assuming a standard state of 1 M. The rate constants (*k*) were obtained from eqn (4), and the computational details are summarized in Table S1 of the SI.^22–28^4
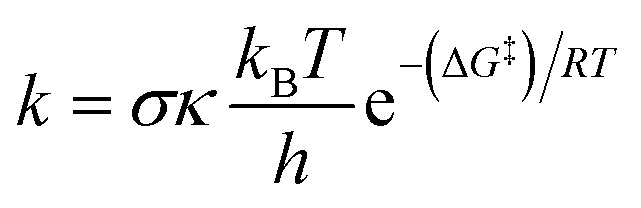
In this expression, *σ* denotes the reaction symmetry number,^29,30^ while *κ* represents the tunneling correction factor estimated using the Eckart barrier model.^31^ The constants *k*_B_ and *h* correspond to the Boltzmann and Planck constants, respectively, and Δ*G*^‡^ refers to the Gibbs free energy of activation.

All quantum chemical calculations were carried out using the M06-2X/6-311++G(d,p) level of theory implemented in the Gaussian 16 program package.^32^ This computational protocol has been widely reported to provide reliable predictions for both thermodynamic and kinetic parameters.^17,33–37^ Solvent effects were incorporated through the SMD continuum solvation model, considering DMSO as a representative polar medium and benzene to describe non-polar environments.^38^ This approach is commonly employed in studies of antioxidant–radical interactions and typically yields rate constants that agree well with experimental measurements, with calculated-to-experimental ratios (*k*_calc_/*k*_exp_) generally ranging from 0.3 to 2.9.^19,20,27,39,40^

## 3. Results and discussion

### 3.1. Tautomerization of HP-136

**Fig. 2:**
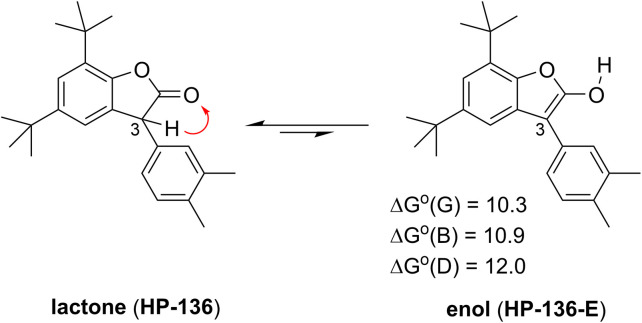
The tautomerization of HP-136 and the relative free energies Δ*G*° (in kcal mol^−1^) compared to the HP-136 conformer (G: the gas phase; B: benzene; D: DMSO).

The tautomerization of HP-136 was investigated in the gas phase and in two solvents, including benzene and DMSO. The calculated relative Gibbs free energies (Δ*G*°) of the tautomeric form with respect to the most stable HP-136 conformer are presented in Fig. 2.

In the gas phase, the enol tautomer was found to be thermodynamically less stable than the reference conformer by 10.3 kcal mol^−1^. A similar trend was observed in benzene, where the relative free energy slightly increased to 10.9 kcal mol^−1^. In the more polar solvent DMSO, the free energy difference further increased to 12.0 kcal mol^−1^. These results indicate that the enol tautomeric form is significantly less favorable than the original HP-136 conformer (lactone) under all investigated conditions. Moreover, the increasing Δ*G*° values from the gas phase to benzene and DMSO suggest that solvent polarity does not stabilize the enol tautomeric structure relative to the parent conformer. Instead, the polar environment slightly enhances the thermodynamic preference for the original HP-136 structure. Overall, the large positive Δ*G*° values (10–12 kcal mol^−1^) imply that the equilibrium strongly favors the lactone HP-136 conformer, and tautomerization is unlikely to occur to a significant extent under normal conditions. Thus, the lactone conformer (HP-136) was used to model the radical scavenging activity in further studies.

### 3.2. The radical scavenging activity of HP-136 in the gas phase

#### 3.2.1. Thermodynamic evaluation

The radical scavenging potential of HP-136 toward HO˙ and HOO˙ radicals was first evaluated in the gas phase, based on calculated Δ*G*° for the FHT and RAF mechanisms at suitable moieties to establish which of these reactions are spontaneous in the thermodynamic sense. The results are summarized in Table 1.

**Table 1:** The calculated values of the Δ*G*° (kcal mol^−1^) for the HP-136 + HO˙/HOO˙ reactions according to the mechanisms

Mechanisms	Positions	HO˙	HOO˙
FHT	C3–H	−42.3	−11.1
	C12–H	−17.2	14.1
	C15–H	−17.8	13.4
	C18–H	−26.3	5.0
	C19–H	−26.7	4.6
RAF	C4	−7.3	
	C5	−12.1	
	C6	−5.7	
	C7	−10.8	
	C8	−12.3	
	C9	−9.5	
	C1′	−9.8	
	C2′	−11.4	
	C3′	−8.2	
	C4′	−8.1	
	C5′	−6.0	
	C6′	−9.1	

For HO˙, all investigated FHT pathways show negative Δ*G*° values, ranging from −17.2 to −42.3 kcal mol^−1^, confirming that hydrogen abstraction is thermodynamically favorable at several positions. The most negative Δ*G*° value is observed at C3–H (Δ*G*° = −42.3 kcal mol^−1^), followed by C18–H and C19–H (−26.3 and −26.7 kcal mol^−1^, respectively), while C12–H and C15–H exhibit Δ*G*° values of −17.2 and −17.8 kcal mol^−1^. The most negative Δ*G*° value at C3–H suggests that this site will also likely to play a major role in the FHT mechanism toward HO˙ in the gas phase.

In case of HOO˙ radicals, only the C3–H pathway has a negative Δ*G*° (−11.1 kcal mol^−1^), whereas hydrogen abstraction at any of the remaining positions is thermodynamically unfavorable, with positive Δ*G*° values ranging from 4.6 to 14.1 kcal mol^−1^. Thus, in the gas phase, radical scavenging of HOO˙ should predominantly occur *via* hydrogen transfer at C3–H.

For the RAF mechanism with HO˙, all addition pathways are thermodynamically spontaneous, with Δ*G*° values between −5.7 and −12.3 kcal mol^−1^. However, the magnitude of Δ*G*° is notably smaller than that observed for the FHT channels. This implies that radical addition makes a smaller contribution than hydrogen transfer to the overall reactivity under gas-phase conditions. Although several carbon atoms exhibit comparable Δ*G*° values, the overall energetic preference remains in favor of the FHT mechanism.

#### 3.2.2. Kinetic evaluation

To gain deeper insight into the radical scavenging efficiency of HP-136 in the gas phase, kinetic parameters including activation Gibbs free energies (Δ*G*^‡^), tunneling corrections (*κ*), Eckart rate constants (*k*_Eck_), branching ratios (*Γ*), and overall rate constants (*k*_overall_) were calculated for both HO˙ and HOO˙ radicals. The results are summarized in Table 2.

**Table 2:** Calculated kinetic data for the reaction between HP-136 and HO˙/HOO˙ radicals in the gas phase: Δ*G*^‡^ in kcal mol^−1^, *Γ* in %, and *k*_Eck_, *k*_overall_ in M^−1^ s^−1^

Mechanisms	Positions	HO˙				HOO˙			
		Δ*G*^‡^	*κ*	*k*_Eck_	*Γ*	Δ*G*^‡^	*κ*	*k*_Eck_	*Γ*
FHT	C3–H	4.8	2.5	4.94 × 10^9^	23.9	21.6	169.6	1.69 × 10^−1^	99.4
	C12–H	5.8	1.8	5.54 × 10^9^	26.9				
	C15–H	7.0	2.0	8.43 × 10^8^	4.1				
	C18–H	6.5	2.4	7.23 × 10^8^	3.5	26.8	842.7	3.55 × 10^−4^	0.2
	C19–H	6.5	2.4	7.83 × 10^8^	3.8	26.0	352.4	5.96 × 10^−4^	0.4
RAF	C4	6.0	1.2	3.01 × 10^8^	1.5				
	C5	6.0	1.1	2.83 × 10^8^	1.4				
	C6	4.8	1.2	2.11 × 10^9^	10.2				
	C7	7.7	1.2	1.57 × 10^7^	0.1				
	C8	5.3	1.2	9.03 × 10^8^	4.4				
	C9	5.2	1.1	1.08 × 10^9^	5.3				
	C1′	5.5	1.2	6.63 × 10^8^	3.2				
	C2′	5.0	1.2	1.57 × 10^9^	7.6				
	C3′	6.4	1.2	1.63 × 10^8^	0.8				
	C4′	6.3	1.2	1.81 × 10^8^	0.9				
	C5′	7.1	1.3	5.18 × 10^7^	0.3				
	C6′	5.7	1.2	4.76 × 10^8^	2.3				
*k*_overall_				2.06 × 10^10^				1.70 × 10^−1^	

In the case of HO˙, the calculated activation barriers for the FHT mechanism are low, ranging from 4.8 to 7.0 kcal mol^−1^, consistent with the strong thermodynamic preference identified previously. The lowest barrier is obtained at C3–H (Δ*G*^‡^ = 4.8 kcal mol^−1^), which aligns well with its highly negative reaction free energy (Δ*G*° = ×42.3 kcal mol^−1^). As anticipated from thermodynamic analysis, this site contributes substantially to the overall reactivity (*Γ* = 23.9%, *k* = 4.94 × 10^9^ M^−1^ s^−1^).

Notably, hydrogen abstraction at C12–H has a slightly higher activation barrier (5.8 kcal mol^−1^) but accounts for the largest branching ratio (26.9%) and a comparable rate constant (5.54 × 10^9^ M^−1^ s^−1^). Together, the C3–H and C12–H pathways account for approximately half of the total HO˙ scavenging activity, confirming that FHT is the dominant mechanism in the gas phase. Although several RAF channels are kinetically accessible, their contributions remain moderate; the most favorable addition at C6 (Δ*G*^‡^ = 4.8 kcal mol^−1^) contributes 10.2%, while other positions account for smaller fractions. This outcome is consistent with the thermodynamic prediction that radical addition, having less favourable thermodynamic parameters than hydrogen transfer, would play a secondary role.

The calculated overall rate constant for the reaction with HO˙ is 2.06 × 10^10^ M^−1^ s^−1^, indicating an extremely rapid radical-trapping process approaching the diffusion-controlled limit. This high reactivity arises from the combination of strong thermodynamics and low activation barriers associated with the FHT mechanism, confirming the excellent intrinsic capacity of HP-136 to neutralize hydroxyl radicals under gas-phase conditions.

In contrast, the reaction with HOO˙ is characterized by substantially higher activation barriers. Hydrogen abstraction at C3–H proceeds with Δ*G*^‡^ = 21.6 kcal mol^−1^, whereas alternative sites such as C18–H and C19–H exhibit barriers above 26 kcal mol^−1^. As predicted from thermodynamic considerations, the C3–H pathway overwhelmingly dominates the kinetics, accounting for 99.4% of the total branching ratio and yielding an overall rate constant of 1.70 × 10^−1^ M^−1^ s^−1^. This value is several orders of magnitude lower than that calculated for HO˙, reflecting the intrinsically lower reactivity of peroxyl-type radicals. The pronounced kinetic disparity between HO˙ and HOO˙ therefore mirrors the fundamental difference in electrophilicity and intrinsic reactivity between hydroxyl and peroxyl radicals.

### 3.3. The radical scavenging activity of HP-136 in the non-polar and polar media

Benzene and DMSO were employed in the present work as simplified model solvents representing non-polar and strongly polar aprotic environments, respectively, in order to probe solvent-dependent mechanistic trends of HP-136 radical scavenging. It should be noted, however, that real polymer matrices are structurally heterogeneous and often characterized by restricted molecular mobility, local microviscosity, and interfacial environments that cannot be fully reproduced by homogeneous liquid-phase solvent models. In particular, oxidative degradation frequently occurs near polymer surfaces exposed to oxygen and moisture, where partially polar and hydrogen-bonding microenvironments may exist. Therefore, the mechanistic trends identified in benzene and DMSO should be interpreted as limiting-case models intended to elucidate intrinsic reactivity patterns rather than as direct representations of practical polymer stabilization conditions.

In benzene, HP-136 is expected to remain predominantly in its neutral form (HA) due to the low polarity and poor ion-stabilizing ability of the medium. In strongly polar aprotic DMSO, deprotonation of HP-136 may become thermodynamically accessible under sufficiently basic conditions or in the presence of proton-accepting species, consistent with the experimentally reported p*K*_a_(DMSO) value of 9.56.^6^

The acidity of HP-136 was independently evaluated using the isodesmic reaction approach.^41,42^ This method is well known to yield substantially higher accuracy than conventional absolute thermodynamic cycle calculations, because systematic errors arising from electronic structure methods and solvation models largely cancel between structurally related species.^43,44^ In the isodesmic scheme (eqn (5)), the p*K*_a_ value of the target compound is determined relative to a reference acid (HRef) with a known experimental p*K*_a_ in the same solvent. The proton-transfer equilibrium and the corresponding p*K*_a_ relationship are expressed as follows:

5 HP-136 + Ref^−^ ⇌ HP-136^−^ + Href

6
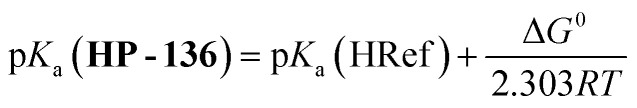
where Δ*G*° is the standard Gibbs free energy change associated with the isodesmic proton-transfer reaction (eqn (5)).

Two reference compounds containing the C–H bond adjacent to a carbonyl group, structurally analogous to that of HP-136, were selected: 2-indanone (p*K*_a_ = 16.9)^45^ and acetophenone (p*K*_a_ = 24.7).^46^ The calculated p*K*_a_ values of HP-136 were 9.57 and 9.70 when referenced to 2-indanone and acetophenone, respectively (Table S2, SI). These values are in excellent agreement with the experimental p*K*_a_ value, thereby confirming the reliability and robustness of the computational methodology employed in this study.

In DMSO solvent, HP-136 undergoes partial ionization according to the following equilibrium:^46,47^HA + DMSO ⇌ A^−^ + DMSOH^+^

The acid dissociation constant in DMSO is defined as:7
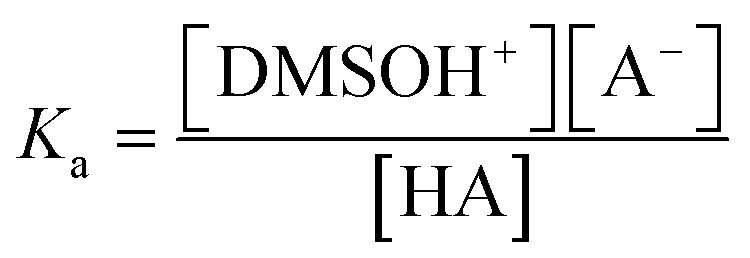


The mole fraction of the deprotonated form, *f* = [A^−^]/[HA]_0_, where [HA]_0_ is the initial concentration of HP-136, is given by:8
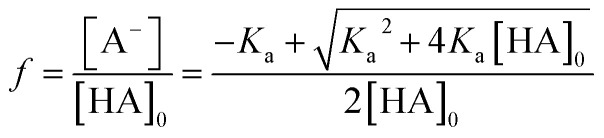
9
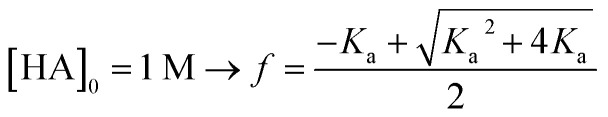


**Fig. 3:**
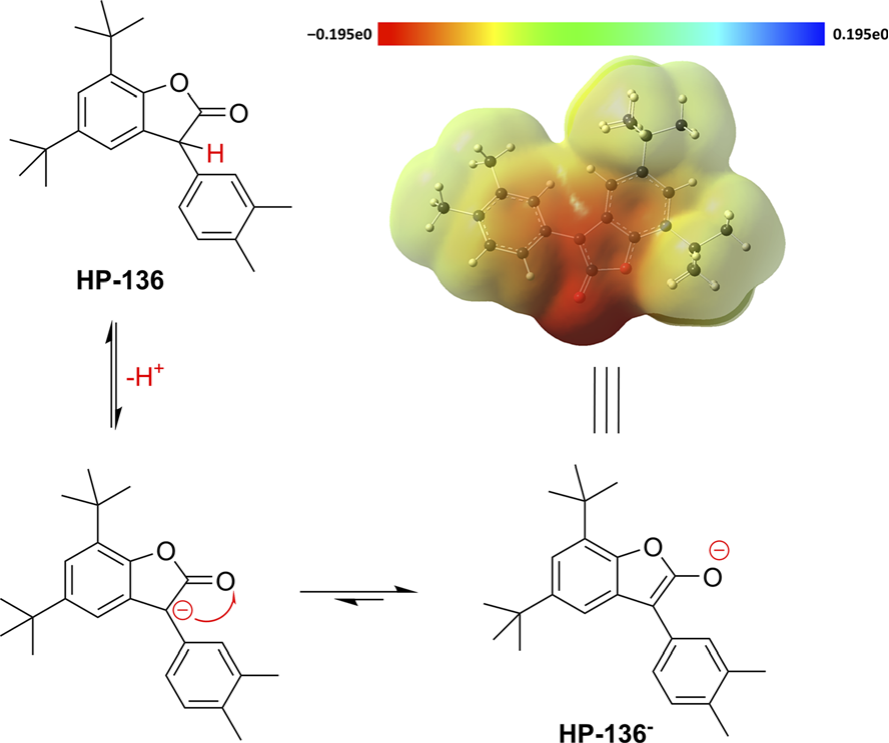
The deprotonation of HP-136 and the electrostatic potential (ESP) map of HP-136^−^.

Using the experimentally determined p*K*_a_ value of 9.56, the mole fraction (*f*) of the deprotonated form, HP-136^−^, was calculated as 1.66 × 10^−5^. Based on the molecular structure of HP-136, deprotonation in DMSO was predicted to preferentially occur at the C3–H position, with a proton affinity value of 23.2 kcal mol^−1^. The resulting negative charge is delocalized between the α-carbon (C3) and the carbonyl oxygen (C=O), leading to the formation of the HP-136^−^ anion. Natural population analysis (NPA) of the HP-136^−^ anion that provides insights into the distribution of partial atomic charges indicated that the additional negative charge is predominantly concentrated on the O2 center. This finding is consistent with the charge distribution pattern inferred from the electrostatic potential (ESP) surface presented in Fig. 3. Thus, in DMSO, the radical-scavenging capacity of HP-136 was assessed through the sequential proton loss-electron transfer (SPLET) mechanism that simplifies to direct single-electron transfer (SET) for the deprotonated form (HP-136^−^), eliminating the normally high activation energy barrier.

Gas-phase calculations indicated that the reaction rate is determined by hydrogen transfer at C3–H and C12–H, together with radical addition at C6. Consequently, solvent effects were evaluated primarily for these kinetically relevant positions. To probe the hydrogen-transfer and radical-addition reactivity of the neutral form, representative oxygen-centered radicals were selected. Hydroxyl radical was included as a highly reactive initiating species capable of non-selective hydrogen abstraction.^48^ Hydroperoxyl radical (HOO˙) represents a typical peroxyl-type propagation species formed under oxidative conditions.^49^ Alkoxyl radicals such as TBO˙ and CMO˙ originate from peroxide decomposition and are closely associated with chain scission and secondary oxidation processes in polymers.^50^ Finally, a 2-cyanoprop-2-yl (AI˙) was considered as a model nitrogen-centered radical with moderate reactivity, allowing comparison with oxygen-centered oxidants.^51^ These radicals therefore provide a mechanistically relevant set to evaluate hydrogen-transfer and addition pathways under conditions related to oxidative degradation.

**Fig. 4:**
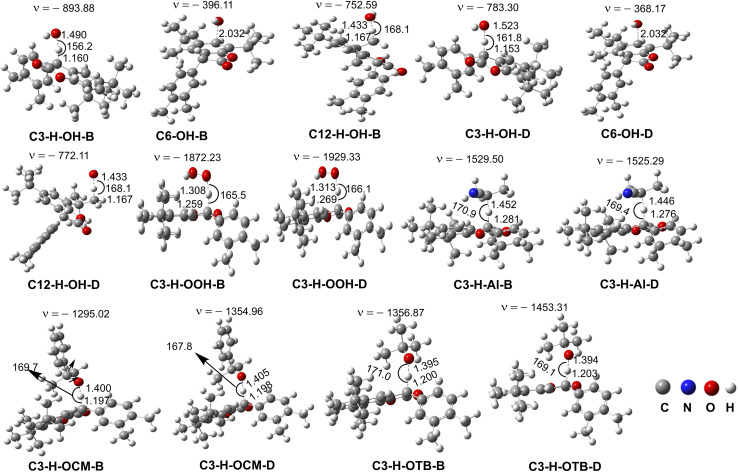
The TS structures of the reactions between the HP-136 and HO˙/HOO˙/TBO˙/CMO˙/AI˙ radicals in benzene.

The calculated kinetic parameters for the reactions of HP-136 with HO˙, HOO˙, TBO˙, CMO˙, and AI˙ radicals in benzene and DMSO are summarized in Table 3, whereas the optimized TS structures are presented in Fig. 4. The detailed SET kinetic parameters are provided in Table S3 of the SI.

**Table 3:** Calculated kinetic data (Δ*G*^‡^ in kcal mol^−1^, *Γ* in %, and *k*_Eck_, *k*_overall_ in M^−1^ s^−1^) for the reactions between HP-136 and HO˙/HOO˙/TBO˙/CMO˙/AI˙ radicals in the benzene and DMSO

Rad.	Mechanism			Benzene				DMSO				
				Δ*G*^‡^	*κ*	*k*_app_	*Γ*	Δ*G*^‡^	*κ*	*k*_app_	*k*_f_	*Γ*
HO˙	HA	FHT	C3–H	6.1	2.3	4.50 × 10^8^	78.4	6.3	1.9	2.30 × 10^8^	2.30 × 10^8^	55.3
			C12–H	8.0	1.8	1.40 × 10^7^	2.4	7.9	1.9	1.60 × 10^8^	1.60 × 10^8^	38.5
		RAF	C6	6.6	1.2	1.10 × 10^8^	19.2	7.4	1.2	2.60 × 10^8^	2.60 × 10^7^	6.2
	A^−^	SET						6.7	1.5^a^	7.50 × 10^7^	1.25 × 10^3^	0
		FHT	C12–H					14.8	4.7	3.90 × 10^3^	6.47 × 10^−2^	0
		RAF	C4					1.2	1.2	1.20 × 10^9^	1.99 × 10^4^	0
			C6					≈0	1.2	1.20 × 10^9^	1.99 × 10^4^	0
	*k*_overall_					5.74 × 10^8^					4.16 × 10^8^	
HOO˙	HA	FHT	C3–H	17.6	53.4	4.40 × 10^1^		20.0	124.0	1.70	1.70	100
TBO˙	HA	FHT	C3–H	10.9	5.4	3.50 × 10^5^		12.3	2.9	1.70 × 10^4^	1.70 × 10^4^	27.5
	A^−^	SET						3.7	6.5^a^	2.70 × 10^9^	4.48 × 10^4^	72.5
	*k*_overall_					3.50 × 10^5^					6.18 × 10^4^	
CMO˙	HA	FHT	C3–H	12.9	2.1	4.70 × 10^3^		13.6	3.6	2.20 × 10^3^	2.20 × 10^3^	3.8
	A^−^	SET						1.1	6.1^a^	3.40 × 10^9^	5.64 × 10^4^	96.2
	*k*_overall_					4.70 × 10^3^					5.86 × 10^4^	
AI˙	HA	FHT	C3–H	19.0	12.2	8.70 × 10^−1^		19.1	8.6	4.90 × 10^−1^	4.90 × 10^−1^	100
	A^−^	SET						30.9	4.2^a^	1.40 × 10^−10^	2.32 × 10^−15^	0
	*k*_overall_					8.70 × 10^−1^					4.90 × 10^−1^	

a *λ*, *k*_f_ = 
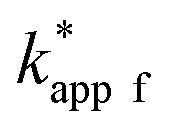
; *f*(HA) = 0.9999834; *f*(A^−^) = 1.66 × 10^−5^; HA = HP-136; A^−^ = HP-136^−^.

A clear solvent-dependent trend can be observed from Table 3. In non-polar benzene, HP-136 remains in its neutral form and the reactions are predominantly governed by hydrogen atom transfer, whereas in polar DMSO the formation of the anionic species enables single electron transfer. Such a mechanistic shift from hydrogen-donation in apolar media to electron-transfer in polar environments has been widely reported for phenolic antioxidants, where solvent polarity stabilizes charged intermediates and lowers activation barriers for SET processes.^52–55^ Consistent with this trend, in benzene, the reaction with HO˙ proceeds predominantly *via* the FHT mechanism at C3–H, with an activation barrier of 6.1 kcal mol^−1^ and a rate constant of 4.50 × 10^8^ M^−1^ s^−1^, accounting for 78.4% of the total branching ratio. Radical addition at C6 remains competitive (Δ*G*^‡^ = 6.6 kcal mol^−1^), contributing 19.2% (*k* = 1.10 × 10^8^ M^−1^ s^−1^), whereas abstraction at C12–H plays only a minor role (2.4%). The overall rate constant in benzene reaches 5.74 × 10^8^ M^−1^ s^−1^, indicating that hydrogen transfer remains the dominant pathway in non-polar media, in agreement with the intrinsic reactivity predicted in the gas phase. In DMSO, despite the exceptionally high apparent rate constants calculated for the RAF pathway at the C6 and C4 positions (*k*_app_ = 1.20 × 10^9^ M^−1^ s^−1^), the anionic form makes a negligible contribution to the HO˙ scavenging activity of HP-136 because of its extremely low mole fraction (*f* = 1.66 × 10^−5^). It is noteworthy that the SET pathway exhibits a high apparent rate constant (*k*_app_ = 7.50 × 10^7^ M^−1^ s^−1^). Nevertheless, this reaction makes a negligible contribution to the overall HO˙ scavenging activity of HP-136 in DMSO because of the extremely low mole fraction of the anionic species. Thus, the HO˙ radical scavenging activity is dominated by the FHT mechanism occurring at the C3–H site (*k*_f_ = 2.30 × 10^8^ M^−1^ s^−1^, *Γ* = 55.3%) and the C12–H site (*k*_f_ = 1.20 × 10^8^ M^−1^ s^−1^, *Γ* = 38.5%), together with the RAF process at the C6 position (*k*_f_ = 2.60 × 10^7^ M^−1^ s^−1^, *Γ* = 38.5%) of the neutral state. The overall rate constant in DMSO was calculated to be *k*_overall_ = 4.16 × 10^9^ M^−1^ s^−1^, which is lower than those reported for typical hydrophilic antioxidants, such as ascorbic acid and Trolox (10^9^–10^10^ M^−1^ s^−1^) toward HO˙ in polar media,^20,56^ These results indicate that HP-136 exhibits a comparatively lower HO˙ scavenging efficiency, although its activity remains within the same order of magnitude as that of widely used dietary antioxidants.

In the case of relatively less reactive radical species, such as HOO˙ and Al˙, the kinetics in benzene are considerably attenuated. The FHT process at the C3–H site is characterized by Δ*G*^‡^ of 17.6 and 19.0 kcal mol^−1^, yielding rate constants of 4.40 × 10^1^ and 8.70 × 10^−1^ M^−1^ s^−1^, respectively. By contrast, the investigated compound displayed pronounced scavenging efficiency toward the TBO˙ radical, with a computed rate constant of 3.50 × 10^5^ M^−1^ s^−1^. This theoretical estimate is in reasonable agreement with the available experimental value (*k*_app_ = 1.35 × 10^5^ M^−1^ s^−1^).^6^ Although only one experimental data point is available for the present system, this agreement provides additional support for the applicability of the computational protocol, which has been extensively validated against experimental kinetic data for a broad range of radical reactions in solution phase.^19,20,27,39,40^ Meanwhile, the antiradical performance against the CMO˙ species is intermediate, with a calculated rate constant of 4.70 × 10^3^ M^−1^ s^−1^.

In DMSO, the HOO˙ antiradical activity was predominantly governed by the FHT mechanism of the neutral species (Δ*G*° = −9.3 kcal mol^−1^, Δ*G*^‡^ = 20 kcal mol^−1^, *k*_f_ = 1.70 M^−1^ s^−1^). The calculated SET thermodynamics for the HOO˙/HOO^−^ couple (Table S3, SI) indicate that the reduction of HOO˙ to HOO^−^ is approximately 24 kcal mol^−1^ less favorable than the reduction of DPPH to DPPH^−^. This result is inconsistent with previous benchmark thermochemical studies, which reported nearly identical electron-acceptor contributions for HOO˙ and DPPH in SET reactions.^21^ Consequently, the calculated SET pathway involving HOO^−^ was not considered in the mechanistic interpretation. This discrepancy most likely arises from the continuum-only treatment of HOO^−^, a small and highly localized anion whose solvation free energy is highly sensitive to specific solvent interactions.^21,57^ A more reliable model chemistry would require a cluster-continuum approach including explicit solvent molecules, which is beyond the scope of the present work. Therefore, the HOO˙ scavenging activity discussed herein is based exclusively on the FHT. A similar trend was observed for the AI˙ radical in DMSO, where the FHT reaction of the neutral form was found to be the dominant pathway determining the radical-scavenging activity of HP-136. In contrast, for the alkoxyl radicals TBO˙ and CMO˙, the calculated overall rate constants increased dramatically in DMSO, reaching *k*_overall_ = 6.18 × 10^4^ and 5.86 × 10^4^ M^−1^ s^−1^, respectively. This enhancement is primarily attributed to the electron-transfer process from the anionic species, with the corresponding SET rate constants being *k*_f_ = 4.48 × 10^4^ and 5.64 × 10^4^ M^−1^ s^−1^, respectively. Consequently, the SET reaction of the anionic form, HP−136^−^, dominates the scavenging activity toward alkoxyl radicals in DMSO, contributing approximately 72.5% and 96.2% to the overall rate constants for TBO˙ and CMO˙, respectively. In contrast, the FHT pathway at the C3–H position accounts for only about 27.5% and 3.8% of the overall reactivity, respectively. The computed results indicated that HP-136 exhibits moderate scavenging activity toward HO˙, TBO˙, and CMO˙ radicals in both nonpolar and polar media. In contrast, the antioxidant shows comparatively low antiradical activity against HOO˙ and AI˙ radicals in the studied environments.

### 3.4. Reactivity of HP-136 toward DPPH in DMSO following the SET mechanism

**Fig. 5:**
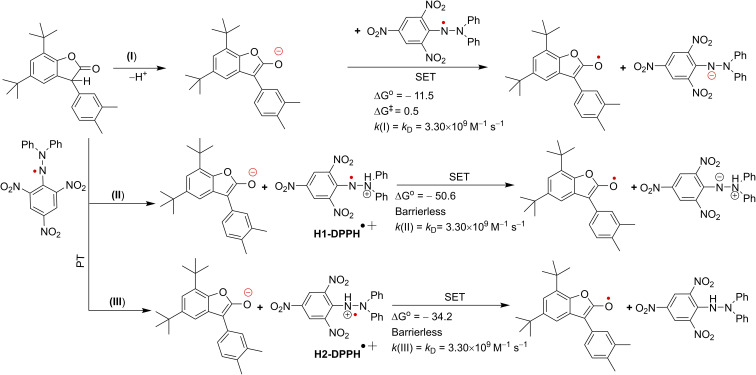
The SET reaction of HP-136 with DPPH in the DMSO.

Previous studies have suggested that the DPPH˙ scavenging activity of HP-136 in DMSO proceeds through a two-step mechanism involving proton transfer (PT) followed by single electron transfer (SET), as illustrated in Scheme II, with an experimentally determined rate constant of *k* = 0.696 M^−1^ s^−1^.^6^ To evaluate the SET step in greater detail, possible electron-transfer reactions were investigated for three states of DPPH, including the neutral DPPH˙ radical (Scheme I), the N1-protonated cation radical (H1-DPPH˙^+^, Scheme II), and the N2-protonated cation radical (H2-DPPH˙^+^, Scheme III). The calculated results are presented in Fig. 5.

It was found that the SET reactions described in Schemes II and III are barrierless, whereas the corresponding activation free energy for Scheme I is only Δ*G*^‡^ = 0.5 kcal mol^−1^. Consequently, the SET rate constants were estimated to be diffusion-limited, with *k*_app_(SET) = *k*_D_, giving a calculated rate constant of *k*_f_ = 5.48 × 10^4^ M^−1^ s^−1^ in DMSO. Although an explicit kinetic evaluation of the proton-transfer step between HP-136 and DPPH was not feasible because of the large size and structural complexity of the bimolecular system, the proton-transfer pathways were calculated to be thermodynamically unfavorable (Δ*G*° = 11.6 kcal mol^−1^).^6^ Accordingly, the diffusion-controlled SET rate constant represents only the intrinsic reactivity of the preformed HP-136^−^ species and should not be interpreted as the overall DPPH scavenging rate. These findings are consistent with the mechanistic interpretation proposed in the previous experimental study.^6^

## 4. Conclusion

A systematic kinetic and mechanistic analysis of HP-136 revealed that its radical-scavenging activity is strongly medium-dependent and mechanistically versatile. In benzene and DMSO solvents, HP-136 predominantly reacts through the FHT mechanism, particularly at the C3–H and C12–H positions, resulting in moderate HO˙ scavenging activity with overall rate constants on the order of 10^8^ M^−1^ s^−1^. In contrast, HP-136 exhibited relatively low scavenging activity toward HOO˙ and AI˙ radicals in both lipid and polar media. The scavenging activity toward TBO˙ and CMO˙ radicals was found to be moderate in benzene and DMSO, with overall rate constants ranging from 10^3^ to 10^5^ M^−1^ s^−1^. The calculated results further indicated that the SET pathway is not the rate-determining step in the DPPH˙, and AI˙ scavenging reactions of HP-136 in DMSO. Nevertheless, this mechanism may make a substantial contribution to the antiradical activity toward CMO˙ and TBO˙ radicals, particularly in polar aprotic media where electron-transfer processes become more favorable.

## Conflicts of interest

There are no conflicts to declare.

## Supplementary Material

RA-OLF-D6RA04239B-s001

## Data Availability

All relevant necessary data to reproduce all results in the paper are within the main text and supplementary information (SI). The Cartesian coordinates, the frequency and energies of transition states for running calculations are also included in the SI. Supplementary information is available. See DOI: https://doi.org/10.1039/d6ra04239b.

## References

[cit1] Hu L., Zhang Y., Zhang M., Chen Q., Zhou Y., Fu J., Zhang D., Pan X. (2025). Environ. Sci. Eur..

[cit2] Oh S., Stache E. E. (2024). Chem. Soc. Rev..

[cit3] Almeida S., Ozkan S., Gonçalves D., Paulo I., Queirós C. S., Ferreira O., Bordado J., Galhano dos Santos R. (2022). Polymers.

[cit4] Ahn Y., Roma G., Colin X. (2022). Macromolecules.

[cit5] Mar'in A., Greci L., Dubs P. (2002). Polym. Degrad. Stab..

[cit6] Zhu X.-Q., Zhou J., Wang C.-H., Li X.-T., Jing S. (2011). J. Phys. Chem. B.

[cit7] Rychlý J., Mosnáčková K., Rychlá L., Fiedlerová A., Kasza G., Nádor A., Osváth Z., Stumphauser T., Szarka G., Czaníková K. (2015). Polym. Degrad. Stab..

[cit8] Meng X., Xin Z., Li Y., Cai Z. (2007). Polym. Degrad. Stab..

[cit9] Font-Sanchis E., Aliaga C., Bejan E. V., Cornejo R., Scaiano J. (2003). J. Org. Chem..

[cit10] Aliaga C., Stuart D. R., Aspée A., Scaiano J. (2005). Org. Lett..

[cit11] Font-Sanchis E., Aliaga C., Focsaneanu K.-S., Scaiano J. (2002). Chem. Commun..

[cit12] Font-Sanchis E., Aliaga C., Cornejo R., Scaiano J. (2003). Org. Lett..

[cit13] Hoa N. T., Vo Q. V. (2023). Chemosphere.

[cit14] Galano A., Raúl Alvarez-Idaboy J. (2019). Int. J. Quantum Chem..

[cit15] Gao Y., Ji Y., Li G., An T. (2016). Water Res..

[cit16] Galano A., Mazzone G., Alvarez-Diduk R., Marino T., Alvarez-Idaboy J. R., Russo N. (2016). Annu. Rev. Food Sci. Technol..

[cit17] Galano A., Alvarez-Idaboy J. R. (2014). J. Comput. Chem..

[cit18] Boulebd H., Mechler A., Hoa N. T., Vo Q. V. (2020). New J. Chem..

[cit19] Galano A., Alvarez-Idaboy J. R. (2013). J. Comput. Chem..

[cit20] Alberto M. E., Russo N., Grand A., Galano A. (2013). Phys. Chem. Chem. Phys..

[cit21] Alvarez-Idaboy J. R. (2026). Comput. Theor. Chem..

[cit22] Evans M. G., Polanyi M. (1935). Trans. Faraday Soc..

[cit23] Eyring H. (1935). J. Phys. Chem..

[cit24] Truhlar D. G., Hase W. L., Hynes J. T. (1983). J. Phys. Chem..

[cit25] Furuncuoglu T., Ugur I., Degirmenci I., Aviyente V. (2010). Macromolecules.

[cit26] Vélez E., Quijano J., Notario R., Pabón E., Murillo J., Leal J., Zapata E., Alarcón G. (2009). J. Phys. Org. Chem..

[cit27] Dzib E., Cabellos J. L., Ortíz-Chi F., Pan S., Galano A., Merino G. (2019). Int. J. Quantum Chem..

[cit28] Dzib E. , Cabellos J. L., Ortiz-Chi F., Pan S., Galano A. and Merino G., Eyringpy 1.0.2, Cinvestav, Mérida, Yucatán, 2018

[cit29] Pollak E., Pechukas P. (1978). J. Am. Chem. Soc..

[cit30] Fernández-Ramos A., Ellingson B. A., Meana-Pañeda R., Marques J. M., Truhlar D. G. (2007). Theor. Chem. Acc..

[cit31] Eckart C. (1930). Phys. Rev..

[cit32] Frisch M. J. , Trucks G. W., Schlegel H. B., Scuseria G. E., Robb M. A., Cheeseman J. R., Scalmani G., Barone V., Mennucci B., Petersson G. A., Nakatsuji H., Caricato M., Li X., Hratchian H. P., Izmaylov A. F., Bloino G. Z. J., Sonnenberg J. L., Hada M., Ehara M., Toyota K., Fukuda R., Hasegawa J., Ishida M., Nakajima T., Honda Y., Kitao O., Nakai H., Vreven T., Montgomery Jr J. A., Peralta J. E., Ogliaro F., Bearpark M., Heyd J. J., Brothers E., Kudin K. N., Staroverov V. N., Keith T., Kobayashi R., Normand J., Raghavachari K., Rendell A., Burant J. C., Iyengar S. S., Tomasi J., Cossi M., Rega N., Millam J. M., Klene M., Knox J. E., Cross J. B., Bakken V., Adamo C., Jaramillo J., Gomperts R., Stratmann R. E., Yazyev O., Austin A. J., Cammi R., Pomelli C., Ochterski J. W., Martin R. L., Morokuma K., Zakrzewski V. G., Voth G. A., Salvador P., Dannenberg J. J., Dapprich S., Daniels A. D., Farkas O., Foresman J. B., Ortiz J. V., Cioslowski J. and Fox D. J., Gaussian 16, Revision B.01, Gaussian, Inc., Wallingford CT, 2016

[cit33] Carreon-Gonzalez M., Vivier-Bunge A., Alvarez-Idaboy J. R. (2019). J. Comput. Chem..

[cit34] Zhao Y., Schultz N. E., Truhlar D. G. (2006). J. Chem. Theory Comput..

[cit35] Zhao Y., Truhlar D. G. (2008). Theor. Chem. Acc..

[cit36] Denis P. A. (2013). Journal of Physical Chemistry.

[cit37] Denis P. A., Yanney M. (2016). RSC Adv..

[cit38] Marenich A. V., Cramer C. J., Truhlar D. G. (2009). J. Phys. Chem. B.

[cit39] Iuga C., Alvarez-Idaboy J. R. l., Russo N. (2012). J. Org. Chem..

[cit40] Vo Q. V., Bay M. V., Nam P. C., Quang D. T., Flavel M., Hoa N. T., Mechler A. (2020). J. Org. Chem..

[cit41] Shen K., Fu Y., Li J.-N., Liu L., Guo Q.-X. (2007). Tetrahedron.

[cit42] Khursan S. L., Ovchinnikov M. Y. (2014). J. Phys. Org. Chem..

[cit43] Casasnovas R., Ortega-Castro J., Frau J., Donoso J., Munoz F. (2014). Int. J. Quantum Chem..

[cit44] Yu H.-Z., Yang Y.-M., Zhang L., Dang Z.-M., Hu G.-H. (2014). Journal of Physical Chemistry.

[cit45] Bordwell F. G. (1988). Acc. Chem. Res..

[cit46] Matthews W. S., Bares J. E., Bartmess J. E., Bordwell F., Cornforth F. J., Drucker G. E., Margolin Z., McCallum R. J., McCollum G. J., Vanier N. R. (1975). J. Am. Chem. Soc..

[cit47] Kolthoff I. M., Chantooni Jr M. K., Bhowmik S. (1968). J. Am. Chem. Soc..

[cit48] Dong C., Fang W., Yi Q., Zhang J. (2022). Chemosphere.

[cit49] Denisov E. T. and Afanas'ev I. B., Oxidation and Antioxidants in Organic Chemistry and Biology, CRC Press, 2005

[cit50] Chatgilialoglu C. and Studer A., Biology and Materials, John Wiley & Sons, 2012

[cit51] Leifert D., Studer A. (2023). Chem. Rev..

[cit52] Trung N. Q., Thong N. M., Cuong D. H., Manh T. D., Hoang L. P., Hien N. K., Nam P. C., Quang D. T., Mechler A., Vo Q. V. (2021). ACS Omega.

[cit53] Nguyen T. Q., Mechler A., Vo Q. V. (2024). RSC Adv..

[cit54] Leopoldini M., Russo N., Toscano M. (2011). Food Chem..

[cit55] Leopoldini M., Marino T., Russo N., Toscano M. (2004). Journal of Physical Chemistry.

[cit56] Shen J., Griffiths P. T., Campbell S. J., Utinger B., Kalberer M., Paulson S. E. (2021). Sci. Rep..

[cit57] Rufino V. C., Pliego Jr J. R. (2021). Phys. Chem. Chem. Phys..

