# Supplementary material for: Solvent effects on the radical scavenging activity of HP-136 benzofuranone: mechanistic and kinetic insights from quantum chemical calculations
Source: RSC Adv. 2026 Jul 15;16(37):40656–64. doi: 10.1039/d6ra04239b (PMC13372134; doi:10.1039/d6ra04239b)
Supplement: Supplementary file 1 [file RA-016-D6RA04239B-s001.pdf]

Supplementary Information (SI) for RSC Advances.
This journal is © The Royal Society of Chemistry 2026

Supporting Information (SI)

Solvent effects on the radical scavenging activity of HP-136 benzofuranone: mechanistic and kinetic insights from quantum chemical calculations

Tran Duc Manh,[1] Vo Thao My,[2] Vo Huynh Ngoc Diep,[2] Truong Le Bich Tram,[3] Mai Van Bay[1], Tran Thanh Diep,[4] Nguyen Quang Trung[5], Adam Mechler[6] and Quan V. Vo[2*]

[1]The University of Danang - University of Sciences and Education, Danang 550000, Vietnam

[2]The University of Danang - University of Technology and Education, Danang 550000, Vietnam.

[3]Department of Science and International Cooperation, The University of Danang, Danang 550000, Vietnam

[4]Center of National Defense – Security Education and Sports, The University of Danang

[5]Quality assurance and Testing center 2, Da Nang 550000, Vietnam.

[6]Department of Biochemistry and Chemistry, La Trobe University, Victoria 3086, Australia.

**Corresponding authors: vvquan@ute.udn.vn;*

Table of Contents

Table S1. The method to calculate rate constant following the conventional transition state theory S3

Table S2: Calculated ΔG° and pKa values of HP-136 obtained from isodesmic reactions using 2-indanone (Href1)[21] and acetophenone (Href1)[22] as reference acids. S5

Table S3: Calculated ΔG^o, ΔE, $\Delta G^‡$ (kcal/mol), the nuclear reorganization energy (λ, in kcal/mol), k, k_{app}, k_D, k_f (in $M^{-1} s^{-1}$) for the SET reactions between BF and $HO^•$/$HOO^•$/$Al^•$/$CMO^•$/$TBO^•$/DPPH in the DMSO S6

Table S4: The Cartesian coordinates, energies of TS of the reaction between BF with $HO^•$/$HOO^•$/$Al^•$/$CMO^•$/$TBO^•$ in the studied media (G: the gas phase; B: benzene; D: DMSO) S7

Table S5: Cartesian coordinates and electronic energies of all optimized species involved in the SET reactions between BF and $HO^•$/$HOO^•$/$Al^•$/$CMO^•$/$TBO^•$/DPPH in the DMSO S51

References S68

Table S1. The method to calculate rate constant following the conventional transition state theory

The rate constant (k) was calculated by using the conventional transition state theory (TST) (at 298.15 K, 1M standard state) according to the equation (1):[1-5]

$$k = \sigma\kappa \frac{k_B T}{h} e^{-(\Delta G^{\neq})/RT} \qquad (1)$$

Where: σ is the reaction symmetry number ,[6,7]

κ contains the tunneling corrections calculated using the Eckart barrier,[8]

k_B is the Boltzmann constant,

h is the Planck constant,

$\Delta G^{\neq}$ is the Gibbs free energy of activation.

The Marcus Theory was used to estimate the reaction barriers of SET reactions.[9-12] The free energy of reaction $\Delta G^{\neq}$ for the SET pathway was computed following the equations (2,3).

$$\Delta G^{\neq}_{SET} = \frac{\lambda}{4}\left(1 + \frac{\Delta G^{0}_{SET}}{\lambda}\right)^2 \qquad (2)$$

$$\lambda \approx \Delta E_{SET} - \Delta G^{0}_{SET} \qquad (3)$$

where ΔG_{SET} is the Gibbs energy of reaction, ΔE_{SET} is the non-adiabatic energy difference between reactants and vertical products for SET.[13,14]

For rate constants that were close to the diffusion limit a correction was applied to yield realistic results[15]. The apparent rate constants (k_{app}) were calculated following the Collins–Kimball theory in the solvents at 298.15K;[16] the steady-state Smoluchowski rate constant (k_D) for an irreversible bimolecular diffusion–controlled reaction was calculated following the literature as corroding to equations (4,5).[15,17]

$$k_{app} = \frac{k_{TST} k_D}{k_{TST} + k_D} \qquad (4)$$

$$k_D = 4\pi R_{AB} D_{AB} N_A \qquad (5)$$

where R_{AB} is the reaction distance, N_A is the Avogadro constant, and $D_{AB} = D_A + D_B$ (D_{AB} is the mutual diffusion coefficient of the reactants A and B),[16,18] where D_A or D_B is estimated using the Stokes–Einstein formulation (6).[19,20]

$$D_{A\ or\ B} = \frac{k_B T}{6\pi \eta a_{A\ or\ B}} \qquad (6)$$

η is the viscosity of the solvents at 298.15 K (i.e. η(benzene) = 6.04×10^{-4} Pa s, η(DMSO) = 1.99×10^{-3} Pa s and *a* is the radius of the solute.

All transition states were characterized by the existence of only one single imaginary frequency. Intrinsic coordinate calculations (IRCs) were performed to ensure that each transition state is connected correctly with the pre-complex and post-complex.

Table S2: Calculated ΔG° and pKa values of HP-136 obtained from isodesmic reactions using 2-indanone (Href1)[21] and acetophenone (Href1)[22] as reference acids.

Reactions	ΔG°	pKa	pKa(ref)
HP-136 + ref1⁻ = HP-136⁻ + Href1	-9.99421	9.57476	16.9
HP-136 + ref2⁻ = HP-136⁻ + Href2	-20.4564	9.706503	24.7

Table S3: Calculated ΔG°, ΔE, ΔG‡ (kcal/mol), the nuclear reorganization energy (*λ*, in kcal/mol), *k*, k_{app}, k_D, k_f (in M^{-1} s^{-1}) for the SET reactions between BF and HO•/HOO•/AI•/CMO•/TBO•/DPPH in the DMSO

Radicals	ΔG°	ΔE	ΔG‡	λ	*k*	k_D	k_{app}	k_f
HO•	-8.0	-6.4	6.7	1.5	7.70E+07	4.00E+09	7.50E+07	1.25E+03
HOO•	12.6	25.9	12.6	13.3	3.60E+03	3.80E+09	3.60E+03	5.98E-02
DPPH•	-11.5	6.1	0.5	17.6	2.50E+12	3.30E+09	3.30E+09	5.48E+04
H1-DPPH•+	-50.6	-44.9	-50.8	5.7	1.09E+50	3.30E+09	3.30E+09	5.48E+04
H2-DPPH•+	-34.2	-25.9	-34.9	8.3	2.31E+38	3.30E+09	3.30E+09	5.48E+04
TBO•	3.3	9.8	3.7	6.5	1.20E+10	3.50E+09	2.70E+09	4.48E+04
CMO•	-0.8	5.3	1.1	6.1	9.00E+11	3.40E+09	3.40E+09	5.64E+04
AI•	18.6	22.9	30.9	4.2	1.40E-10	3.50E+09	1.40E-10	2.32E-15

k_f = f*k_{app}; f = 1.66E-05 (f is the equilibrium molar fraction of HP-136⁻ in DMSO, calculated from the experimental pKa (9.56) assuming acid–base equilibrium at the standard state (1 M))

Table S4: The Cartesian coordinates, energies of TS of the reaction between BF with HO•/HOO•/AI•/CMO•/TBO• in the studied media (G: the gas phase; B: benzene; D: DMSO)

Cartesian Coordinates				Energy	
BF-AL-TS-D					
Atom	X	Y	Z	Electronic Energy (EE)	-1293.63
O	0.649516	-2.45227	0.482454	Zero-point Energy Correction	0.563098
O	-1.34928	-3.2678	1.082871	Thermal Correction to Energy	0.592401
C	3.533909	-2.07392	-0.23705	Thermal Correction to Enthalpy	0.593345
C	2.505155	-0.95059	-0.10139	Thermal Correction to Free Energy	0.504743
C	2.292658	2.928059	-0.20925		
C	0.217034	-0.20185	0.361193		
C	1.899691	1.451917	-0.08994		
C	1.178731	-1.20006	0.216338		
C	-1.01713	-0.85074	0.847998		
C	2.819829	0.41047	-0.25554		
C	0.575919	1.130929	0.229643		
C	4.917166	-1.53485	-0.61363		
C	3.084497	-3.05452	-1.3334		
C	3.6563	-2.81606	1.105354		
C	-0.67319	-2.31697	0.827479		
C	2.041035	3.620595	1.141097		
C	1.433861	3.600796	-1.29273		
C	3.766634	3.112113	-0.58078		
C	-2.39597	-0.4548	0.452517		
C	-2.62199	0.539881	-0.50234		
C	-3.49611	-1.07559	1.050114		
C	-3.91005	0.924102	-0.86621		
C	-5.01218	0.300393	-0.258		
C	-4.78248	-0.69588	0.691171		
C	-4.11666	2.000621	-1.89796		
C	-6.41449	0.693307	-0.63483		
H	-0.95516	-0.59803	2.101978		
H	3.840456	0.662693	-0.50394		
H	-0.15825	1.908624	0.404075		
H	5.611447	-2.37301	-0.71131		
H	4.900352	-1.00447	-1.56954		
H	5.315339	-0.86266	0.151338		
H	2.998732	-2.54352	-2.29625		
H	3.822394	-3.85521	-1.4377		
H	2.122096	-3.5106	-1.09807		
H	4.41412	-3.60093	1.025935		
H	3.960842	-2.12898	1.900188		
H	2.714521	-3.28502	1.393406		
H	2.318215	4.677154	1.078891		
H	0.99174	3.564736	1.440099		
H	2.638575	3.156744	1.931239		

H	0.369346	3.553096	-1.05107
H	1.706602	4.655979	-1.3859
H	1.584992	3.120802	-2.2636
H	3.988973	4.17941	-0.65737
H	4.432	2.690119	0.17741
H	4.003171	2.653469	-1.54497
H	-1.77911	1.012788	-0.99366
H	-3.34294	-1.85834	1.782995
H	-5.63113	-1.18424	1.15898
H	-3.16775	2.30275	-2.34254
H	-4.77692	1.661136	-2.70061
H	-4.57922	2.887174	-1.45338
H	-6.56614	1.771535	-0.53418
H	-6.62907	0.43503	-1.67655
H	-7.14539	0.18566	-0.00458
C	-0.69168	-0.19703	3.472618
C	-1.77842	-0.89479	4.250608
H	-2.76923	-0.54148	3.963072
H	-1.71899	-1.97352	4.08662
H	-1.6493	-0.71128	5.323918
C	0.722819	-0.69703	3.644829
H	0.764493	-1.77067	3.440167
H	1.418943	-0.17773	2.982971
H	1.053035	-0.54406	4.678687
C	-0.84063	1.215754	3.317117
N	-0.97026	2.347645	3.123244

BF-C1'-OH-TS-G

Atom	X	Y	Z		
O	0.493369	-2.18428	0.678469	Electronic Energy (EE)	-1158.66
O	-1.42245	-2.87572	1.611056	Zero-point Energy Correction	0.496552
C	3.154714	-1.98397	-0.67261	Thermal Correction to Energy	0.524579
C	2.331772	-0.79438	-0.17874	Thermal Correction to Enthalpy	0.525523
C	2.474075	3.082465	-0.03636	Thermal Correction to Free Energy	0.438679
C	0.328286	0.098767	0.905265		
C	1.99671	1.636896	0.134819		
C	1.101471	-0.95646	0.442955		
C	-0.92383	-0.47091	1.503781		
C	2.742087	0.541425	-0.31228		
C	0.765129	1.400994	0.757792		
C	4.468526	-1.53531	-1.3203		
C	2.344227	-2.76772	-1.72014		
C	3.492416	-2.90146	0.51594		
C	-0.708	-1.9776	1.302043		
C	2.587428	3.747049	1.346127		

C	1.451062	3.853004	-0.88873
C	3.8389	3.17161	-0.72581
C	-2.23893	-0.04306	0.880572
C	-2.29825	0.362655	-0.47638
C	-3.44514	-0.44741	1.501648
C	-3.49923	0.478107	-1.15074
C	-4.69962	0.133227	-0.48915
C	-4.64587	-0.32972	0.82992
C	-3.53081	0.956015	-2.57857
C	-6.01651	0.246465	-1.2032
H	-0.98797	-0.28515	2.579608
H	3.692658	0.726689	-0.79053
H	0.13591	2.205829	1.117562
H	5.019512	-2.41711	-1.65531
H	4.293966	-0.89997	-2.19256
H	5.103538	-0.9936	-0.61448
H	2.099169	-2.12744	-2.57165
H	2.934527	-3.61236	-2.08647
H	1.41717	-3.15725	-1.29897
H	4.093665	-3.74678	0.169603
H	4.069061	-2.35701	1.268032
H	2.591064	-3.29484	0.986358
H	2.924353	4.782106	1.239406
H	1.627767	3.756475	1.866632
H	3.306827	3.212941	1.97189
H	0.464316	3.859897	-0.4208
H	1.772447	4.890654	-1.01656
H	1.355158	3.397983	-1.87812
H	4.129323	4.220847	-0.81866
H	4.615199	2.660819	-0.15018
H	3.812354	2.743354	-1.73125
H	-1.37246	0.623705	-0.97846
H	-3.41175	-0.82254	2.517396
H	-5.56672	-0.6123	1.327864
H	-2.52336	1.15734	-2.94207
H	-3.98804	0.210917	-3.23504
H	-4.11672	1.874323	-2.67519
H	-6.19303	1.270044	-1.54601
H	-6.03616	-0.39346	-2.09021
H	-6.8418	-0.0448	-0.55409
O	-2.13323	1.778691	1.792816
H	-2.9849	2.142378	1.509721

BF-C12-OH-TS-B

Atom	X	Y	Z	Electronic Energy (EE)	-1158.68

O	0.57501	-2.29644	0.627837	Zero-point Energy Correction	0.49138
O	-1.32115	-3.07713	1.520396	Thermal Correction to Energy	0.519895
C	3.258489	-1.9749	-0.66203	Thermal Correction to Enthalpy	0.520839
C	2.373266	-0.82535	-0.18058	Thermal Correction to Free Energy	0.430654
C	2.354123	3.052437	-0.01834		
C	0.310717	-0.02689	0.860611		
C	1.929712	1.587479	0.132124		
C	1.137932	-1.04493	0.411966		
C	-0.93397	-0.64804	1.428803		
C	2.732505	0.526039	-0.29797		
C	0.693197	1.292538	0.720589		
C	4.570721	-1.47417	-1.2723		
C	2.507726	-2.79392	-1.73194		
C	3.59171	-2.89151	0.515434		
C	-0.64765	-2.14177	1.227922		
C	2.41113	3.710566	1.370236		
C	1.324641	3.791378	-0.88958		
C	3.730688	3.197663	-0.67337		
C	-2.24703	-0.25289	0.780148		
C	-2.30662	0.071917	-0.57539		
C	-3.4211	-0.25444	1.524402		
C	-3.51156	0.393907	-1.19525		
C	-4.6963	0.392992	-0.43783		
C	-4.62962	0.067088	0.914919		
C	-3.54515	0.73928	-2.65999		
C	-6.01159	0.741367	-1.08067		
H	-1.00685	-0.49033	2.509569		
H	3.689133	0.751893	-0.74551		
H	0.032642	2.08258	1.061483		
H	5.164391	-2.33315	-1.59565		
H	4.392216	-0.84599	-2.14871		
H	5.161059	-0.9103	-0.54621		
H	2.257305	-2.15632	-2.58436		
H	3.143626	-3.60833	-2.09		
H	1.58716	-3.22655	-1.33935		
H	4.264471	-3.701	0.222115		
H	4.15399	-2.256	1.316913		
H	2.718805	-3.29268	1.030874		
H	2.718135	4.756533	1.27805		
H	1.438896	3.690578	1.867974		
H	3.130855	3.196935	2.013199		
H	0.326148	3.76519	-0.44691		
H	1.612865	4.840463	-1.00449		
H	1.264686	3.3421	-1.88476		
H	3.982644	4.258247	-0.75251		
H	4.513247	2.713706	-0.08281		

H	3.746249	2.777648	-1.68286
H	-1.39381	0.079477	-1.1642
H	-3.39466	-0.51058	2.57821
H	-5.54064	0.065946	1.504469
H	-2.54475	0.704383	-3.09269
H	-4.18029	0.042742	-3.2152
H	-3.95131	1.742111	-2.82151
H	-5.99564	1.754564	-1.49315
H	-6.24031	0.062413	-1.90728
H	-6.82695	0.682545	-0.35889
O	5.087449	-1.56821	2.158176
H	5.812686	-2.20934	2.073433

BF-C12-OH-TS-G

Atom	X	Y	Z		
O	0.585732	-2.3045	0.661002	Electronic Energy (EE)	-1158.65
O	-1.30396	-3.06018	1.599487	Zero-point Energy Correction	0.491543
C	3.260437	-2.0135	-0.64021	Thermal Correction to Energy	0.520241
C	2.377626	-0.85172	-0.18689	Thermal Correction to Enthalpy	0.521186
C	2.373988	3.026075	-0.08946	Thermal Correction to Free Energy	0.42862
C	0.318169	-0.02945	0.838945		
C	1.943794	1.5662	0.083703		
C	1.143062	-1.05813	0.411354		
C	-0.92433	-0.63586	1.422218		
C	2.741669	0.494936	-0.32875		
C	0.707158	1.285399	0.677134		
C	4.569169	-1.52842	-1.2715		
C	2.503314	-2.86691	-1.67861		
C	3.602378	-2.88539	0.569089		
C	-0.63529	-2.1373	1.268771		
C	2.430619	3.705711	1.28924		
C	1.349927	3.754113	-0.97697		
C	3.753432	3.15422	-0.74306		
C	-2.24411	-0.27063	0.767243		
C	-2.29796	0.262254	-0.51906		
C	-3.43373	-0.51338	1.446415		
C	-3.51324	0.561103	-1.13247		
C	-4.71075	0.318002	-0.44207		
C	-4.64953	-0.21961	0.842126		
C	-3.53999	1.139136	-2.5231		
C	-6.03732	0.634384	-1.08024		
H	-0.99883	-0.44573	2.497953		
H	3.700447	0.708709	-0.77689		
H	0.048785	2.082797	1.004841		
H	5.156199	-2.39529	-1.58457		

H	4.381579	-0.91657	-2.1573
H	5.165077	-0.9557	-0.55767
H	2.252962	-2.25585	-2.54979
H	3.136887	-3.69364	-2.01084
H	1.583504	-3.28121	-1.26636
H	4.292301	-3.69097	0.307784
H	4.151533	-2.19311	1.332629
H	2.730892	-3.27624	1.093829
H	2.741848	4.748588	1.181604
H	1.456927	3.695618	1.783586
H	3.145809	3.196358	1.939609
H	0.350344	3.736497	-0.53708
H	1.641447	4.800067	-1.10778
H	1.29422	3.286234	-1.96329
H	4.010666	4.211733	-0.83924
H	4.529719	2.674969	-0.14125
H	3.767632	2.715931	-1.74438
H	-1.37529	0.454639	-1.05744
H	-3.40938	-0.94208	2.442233
H	-5.5721	-0.4113	1.379828
H	-2.52967	1.270679	-2.91085
H	-4.08636	0.487451	-3.21075
H	-4.03999	2.1117	-2.53774
H	-6.11356	1.696387	-1.33036
H	-6.17169	0.075509	-2.01064
H	-6.86145	0.383384	-0.41232
O	4.992437	-1.33059	2.092799
H	5.610119	-2.01201	2.403058

BF-C15-OH-TS-G

Atom	X	Y	Z		
O	0.577364	-2.27109	0.683756	Zero-point Energy Correction	0.491502
O	-1.32331	-3.04244	1.587459	Thermal Correction to Energy	0.520105
C	3.281259	-1.95747	-0.5613	Thermal Correction to Enthalpy	0.521049
C	2.372601	-0.80477	-0.13667	Thermal Correction to Free Energy	0.429695
C	2.319431	3.071419	-0.0724		
C	0.287183	0.000173	0.854822		
C	1.896504	1.609332	0.101827		
C	1.12893	-1.0212	0.442935		
C	-0.95705	-0.61599	1.423528		
C	2.716113	0.546283	-0.28817		
C	0.66019	1.319546	0.68851		
C	4.595907	-1.45029	-1.16227		
C	2.56291	-2.81277	-1.61992		
C	3.61528	-2.82106	0.668134		

Electronic Energy (EE): -1158.65

C	-0.65431	-2.11502	1.270324
C	2.474631	3.710062	1.307753
C	1.234254	3.839526	-0.85357
C	3.645804	3.218017	-0.8253
C	-2.27108	-0.25751	0.754959
C	-2.31418	0.238738	-0.54657
C	-3.46599	-0.47147	1.434056
C	-3.52355	0.52859	-1.17521
C	-4.7267	0.315072	-0.48436
C	-4.67645	-0.18551	0.815007
C	-3.53874	1.064636	-2.58271
C	-6.0474	0.622851	-1.13873
H	-1.04419	-0.42892	2.498861
H	3.676487	0.773002	-0.72615
H	-0.01229	2.109027	1.006854
H	5.210471	-2.30732	-1.44714
H	4.425833	-0.84951	-2.05968
H	5.165065	-0.85448	-0.44404
H	2.322541	-2.21076	-2.5002
H	3.214856	-3.63253	-1.93436
H	1.640147	-3.24226	-1.22922
H	4.279282	-3.63842	0.373299
H	4.124318	-2.22305	1.428242
H	2.71721	-3.25435	1.108913
H	2.815812	4.746577	1.25179
H	1.585552	3.630744	1.935702
H	3.300194	3.098737	1.871091
H	0.271679	3.826533	-0.33949
H	1.53278	4.882888	-0.98539
H	1.098305	3.391788	-1.84143
H	3.87807	4.279326	-0.94469
H	4.46677	2.753348	-0.27567
H	3.585936	2.77456	-1.82226
H	-1.38687	0.407445	-1.08511
H	-3.45034	-0.87206	2.441702
H	-5.60333	-0.35442	1.352946
H	-2.52535	1.179512	-2.96773
H	-4.0835	0.394753	-3.25395
H	-4.0343	2.03843	-2.63019
H	-6.11493	1.678061	-1.41846
H	-6.18053	0.039381	-2.05407
H	-6.87696	0.396027	-0.4689
O	4.454929	2.463936	2.379375
H	4.885645	3.254641	2.741889

BF-C18-OH-TS-G

Atom	X	Y	Z		
O	0.582016	-2.2745	0.705874	Electronic Energy (EE)	-1158.65
O	-1.32866	-3.00771	1.620022	Zero-point Energy Correction	0.49261
C	3.284405	-2.01301	-0.55255	Thermal Correction to Energy	0.520758
C	2.388401	-0.84225	-0.15005	Thermal Correction to Enthalpy	0.521702
C	2.344557	3.039941	-0.21058	Thermal Correction to Free Energy	0.432827
C	0.305639	0.003462	0.811736		
C	1.930754	1.584063	0.026069		
C	1.14271	-1.03474	0.43233		
C	-0.94696	-0.59015	1.385618		
C	2.741503	0.503218	-0.33554		
C	0.68933	1.315368	0.6141		
C	4.601689	-1.53335	-1.17008		
C	2.553194	-2.88274	-1.59069		
C	3.613973	-2.85535	0.692672		
C	-0.65089	-2.09486	1.279505		
C	2.364306	3.790397	1.131814		
C	1.327188	3.705745	-1.15329		
C	3.733977	3.156662	-0.84492		
C	-2.25386	-0.25024	0.692083		
C	-2.28457	0.308065	-0.58204		
C	-3.45903	-0.54144	1.327349		
C	-3.49341	0.579663	-1.22796		
C	-4.70775	0.29447	-0.57929		
C	-4.6654	-0.26917	0.694686		
C	-3.48813	1.21459	-2.57621		
C	-6.02284	0.590544	-1.25031		
H	-1.04652	-0.37033	2.453736		
H	3.699969	0.709916	-0.78824		
H	0.020989	2.119372	0.903357		
H	5.205956	-2.40285	-1.43839		
H	4.434556	-0.95067	-2.07975		
H	5.181685	-0.92842	-0.46822		
H	2.313654	-2.2961	-2.48141		
H	3.196338	-3.7142	-1.89202		
H	1.628806	-3.2965	-1.1872		
H	4.26592	-3.68705	0.411699		
H	4.135418	-2.24944	1.438194		
H	2.713053	-3.26792	1.147255		
H	2.659359	4.831698	0.975715		
H	1.3824	3.788842	1.609604		
H	3.076619	3.329644	1.820692		
H	0.319377	3.695581	-0.73214		
H	1.605166	4.747991	-1.33281		
H	1.297037	3.187526	-2.11516		

H	3.978742	4.211888	-0.98669
H	4.506272	2.718042	-0.20777
H	3.772931	2.67223	-1.82392
H	-1.3542	0.537202	-1.09172
H	-3.45177	-0.9918	2.313836
H	-5.59637	-0.49732	1.202482
H	-2.49814	1.241765	-3.02963
H	-4.21862	0.788387	-3.26544
H	-3.81836	2.324565	-2.47909
H	-6.06733	1.628222	-1.59162
H	-6.16562	-0.04755	-2.12759
H	-6.85452	0.413306	-0.56842
O	-4.22296	3.601977	-1.88226
H	-3.97846	3.353567	-0.97505

BF-C18-OOH-TS-G

Atom	X	Y	Z		
				Electronic Energy (EE)	-1233.8
O	0.441331	-2.23084	0.092553	Zero-point Energy Correction	0.497348
O	-1.62605	-2.97831	0.51306	Thermal Correction to Energy	0.526009
C	3.23717	-1.86873	-0.92192	Thermal Correction to Enthalpy	0.526953
C	2.359315	-0.75335	-0.35575	Thermal Correction to Free Energy	0.437015
C	2.451329	3.07208	0.305273		
C	0.246083	-0.0015	0.617276		
C	1.969384	1.619454	0.235917		
C	1.072408	-0.98998	0.105009		
C	-1.05068	-0.64417	1.026816		
C	2.768792	0.586482	-0.26401		
C	0.679672	1.306846	0.682834		
C	4.613219	-1.34646	-1.3467		
C	2.5504	-2.48068	-2.15596		
C	3.442949	-2.95254	0.151069		
C	-0.83796	-2.08563	0.543822		
C	2.406696	3.553467	1.765549		
C	1.526394	3.953664	-0.55116		
C	3.884398	3.237923	-0.20972		
C	-2.32395	-0.07628	0.449855		
C	-2.53759	-0.14689	-0.92279		
C	-3.32446	0.442807	1.261424		
C	-3.75902	0.224887	-1.49528		
C	-4.78046	0.738554	-0.6607		
C	-4.53046	0.862117	0.699223		
C	-3.99087	-0.07667	-2.90466		
C	-6.12156	1.114416	-1.23088		
H	-1.13467	-0.68986	2.118349		

H	3.763211	0.828361	-0.60869
H	0.0168	2.07324	1.069828
H	5.203519	-2.17785	-1.73842
H	4.535339	-0.59346	-2.13522
H	5.16113	-0.91637	-0.50416
H	2.398305	-1.72012	-2.92614
H	3.181774	-3.26914	-2.57473
H	1.584166	-2.91796	-1.90335
H	4.081013	-3.74662	-0.24629
H	3.931439	-2.5309	1.033394
H	2.496539	-3.39914	0.456724
H	2.750934	4.589385	1.830319
H	1.394508	3.510814	2.173127
H	3.051802	2.935665	2.395198
H	0.493315	3.914794	-0.19944
H	1.857013	4.995298	-0.51065
H	1.540454	3.627518	-1.59403
H	4.175903	4.288061	-0.13357
H	4.594995	2.651544	0.378492
H	3.972966	2.94357	-1.2586
H	-1.76126	-0.5469	-1.56999
H	-3.17782	0.501549	2.33451
H	-5.3058	1.261408	1.344152
H	-3.09985	-0.13433	-3.52657
H	-4.29948	-1.35769	-2.85699
H	-4.83751	0.397328	-3.39237
H	-6.02649	1.888357	-1.99698
H	-6.60309	0.249509	-1.69565
H	-6.78189	1.489879	-0.44928
H	-3.22356	-2.85498	-1.15275
O	-4.35351	-2.55356	-2.62749
O	-3.11771	-2.8649	-2.11881

BF-C19-OH-TS-G

Atom	X	Y	Z		
O	0.563855	-2.28497	0.698306	Electronic Energy (EE)	-1158.65
O	-1.335	-3.04382	1.616087	Zero-point Energy Correction	0.492645
C	3.257006	-1.98814	-0.57195	Thermal Correction to Energy	0.520792
C	2.355476	-0.82909	-0.14842	Thermal Correction to Enthalpy	0.521736
C	2.287109	3.053072	-0.1522	Thermal Correction to Free Energy	0.432785
C	0.272244	-0.01061	0.835691		
C	1.882718	1.591349	0.06385		
C	1.114328	-1.03786	0.438055		
C	-0.97265	-0.62038	1.409434		
C	2.698707	0.521134	-0.31701		

C	0.645887	1.306443	0.653929
C	4.567562	-1.4911	-1.19005
C	2.525758	-2.84826	-1.61802
C	3.599169	-2.84537	0.659615
C	-0.66644	-2.12186	1.282367
C	2.314231	3.781062	1.202376
C	1.258541	3.72952	-1.07498
C	3.670462	3.187688	-0.79616
C	-2.28671	-0.28314	0.728907
C	-2.32762	0.271177	-0.54922
C	-3.48301	-0.57376	1.380493
C	-3.53594	0.545408	-1.18687
C	-4.74046	0.246746	-0.52561
C	-4.69282	-0.30598	0.756439
C	-3.55179	1.154732	-2.56406
C	-6.04989	0.558291	-1.16367
H	-1.06725	-0.4153	2.480896
H	3.653429	0.740412	-0.77169
H	-0.02676	2.101552	0.957346
H	5.175985	-2.3527	-1.474
H	4.391363	-0.89682	-2.09045
H	5.147577	-0.89226	-0.483
H	2.27751	-2.25102	-2.49925
H	3.172576	-3.6713	-1.93426
H	1.606281	-3.27355	-1.21529
H	4.254889	-3.66883	0.363512
H	4.120895	-2.2464	1.410516
H	2.703566	-3.2701	1.113478
H	2.602471	4.82647	1.061527
H	1.336405	3.765774	1.688243
H	3.034783	3.312646	1.877352
H	0.254991	3.708079	-0.64431
H	1.531036	4.77563	-1.2402
H	1.221875	3.226223	-2.04454
H	3.908294	4.24638	-0.92325
H	4.450503	2.743592	-0.17242
H	3.703903	2.71876	-1.78291
H	-1.39951	0.501614	-1.06216
H	-3.46503	-1.02028	2.368374
H	-5.62166	-0.53549	1.267901
H	-2.5432	1.417281	-2.88363
H	-3.96541	0.454499	-3.29602
H	-4.17128	2.055208	-2.58695
H	-6.20427	1.709304	-1.18441
H	-6.10279	0.276783	-2.21662
H	-6.89856	0.153866	-0.61377

O	-6.00103	3.14375	-0.94238
H	-5.40319	3.019985	-0.18637

BF-C19-OOH-TS-G

Atom	X	Y	Z	Electronic Energy (EE)	-1233.8
O	0.323392	-2.22454	0.600431	Zero-point Energy Correction	0.497034
O	-1.63026	-2.90333	1.464749	Thermal Correction to Energy	0.525957
C	3.015563	-2.03333	-0.69586	Thermal Correction to Enthalpy	0.526901
C	2.196507	-0.84349	-0.19691	Thermal Correction to Free Energy	0.435357
C	2.376032	3.030637	-0.00446		
C	0.183703	0.053684	0.861539		
C	1.879659	1.588639	0.141592		
C	0.953194	-1.00335	0.39999		
C	-1.09115	-0.50435	1.424954		
C	2.622263	0.489711	-0.30276		
C	0.633453	1.352873	0.733986		
C	4.347004	-1.5884	-1.30903		
C	2.216129	-2.78766	-1.77315		
C	3.317884	-2.97555	0.482839		
C	-0.89298	-2.01272	1.197275		
C	2.464273	3.682391	1.385976		
C	1.384647	3.820344	-0.87633		
C	3.758791	3.109507	-0.65881		
C	-2.38013	-0.03481	0.784213		
C	-2.4163	0.325613	-0.56468		
C	-3.56105	-0.02277	1.522403		
C	-3.60099	0.698131	-1.1891		
C	-4.79619	0.728261	-0.43261		
C	-4.75138	0.349162	0.917393		
C	-3.61578	1.046501	-2.65345		
C	-6.0751	1.029105	-1.06764		
H	-1.1555	-0.35953	2.508015		
H	3.583607	0.671495	-0.75998		
H	0.014146	2.17185	1.083656		
H	4.894834	-2.47058	-1.64778		
H	4.197419	-0.93708	-2.17411		
H	4.973748	-1.06618	-0.58144		
H	1.994292	-2.12925	-2.61713		
H	2.804321	-3.6315	-2.14414		
H	1.277385	-3.17566	-1.3772		
H	3.914942	-3.82213	0.13289		
H	3.887009	-2.45231	1.255561		
H	2.402788	-3.36612	0.928488		
H	2.818977	4.712854	1.29596		

H	1.492387	3.704902	1.883316
H	3.159364	3.133111	2.025774
H	0.386192	3.840044	-0.43429
H	1.723275	4.854043	-0.98899
H	1.305965	3.373411	-1.87062
H	4.063478	4.15596	-0.73387
H	4.514468	2.584002	-0.0693
H	3.752099	2.692099	-1.66896
H	-1.49691	0.311444	-1.14266
H	-3.54998	-0.32082	2.564769
H	-5.67214	0.344675	1.49004
H	-2.62865	0.908571	-3.0943
H	-4.32735	0.416595	-3.19457
H	-3.91432	2.087152	-2.80907
H	-6.06191	1.713725	-1.91222
H	-6.36395	-0.1132	-1.64514
H	-6.91278	1.183476	-0.39176
H	-5.00554	-2.02832	-0.9959
O	-6.36863	-1.27504	-2.03012
O	-5.97151	-1.98864	-0.92674

BF-C2'-OH-TS-G

Atom	X	Y	Z	Electronic Energy (EE)	-1158.66
O	0.60941	-2.30173	0.760124	Zero-point Energy Correction	0.496866
O	-1.27616	-3.03208	1.717712	Thermal Correction to Energy	0.524936
C	3.311414	-2.05281	-0.49964	Thermal Correction to Enthalpy	0.52588
C	2.405125	-0.87813	-0.13209	Thermal Correction to Free Energy	0.438464
C	2.323051	3.001239	-0.2832		
C	0.311254	-0.02299	0.800848		
C	1.930751	1.546285	-0.0107		
C	1.158745	-1.06312	0.449546		
C	-0.93577	-0.61587	1.391159		
C	2.749542	0.465255	-0.35055		
C	0.692092	1.28788	0.587435		
C	4.624619	-1.57982	-1.131		
C	2.589969	-2.96047	-1.51169		
C	3.648804	-2.85493	0.76981		
C	-0.6164	-2.11951	1.337012		
C	2.361273	3.775734	1.045031		
C	1.271381	3.635554	-1.21092		
C	3.695189	3.121897	-0.95241		
C	-2.25737	-0.3151	0.70402		
C	-2.3324	0.614298	-0.36167		
C	-3.42381	-0.9041	1.158802		
C	-3.56156	0.84888	-1.0305		

C	-4.72712	0.243505	-0.56195
C	-4.63887	-0.62424	0.531098
C	-3.59149	1.806842	-2.18507
C	-6.05187	0.513666	-1.22519
H	-1.03675	-0.35649	2.451255
H	3.705928	0.668408	-0.80981
H	0.01191	2.085701	0.861676
H	5.236467	-2.45137	-1.37473
H	4.452032	-1.02426	-2.05646
H	5.199119	-0.94997	-0.44684
H	2.343981	-2.40253	-2.41892
H	3.241703	-3.79378	-1.78883
H	1.669911	-3.37191	-1.09601
H	4.308954	-3.68848	0.514142
H	4.163587	-2.22185	1.497099
H	2.751523	-3.26222	1.236336
H	2.638255	4.818201	0.864195
H	1.388619	3.767183	1.541055
H	3.09484	3.337064	1.726216
H	0.274889	3.614827	-0.76298
H	1.5296	4.678786	-1.41433
H	1.228806	3.100582	-2.16363
H	3.921432	4.176482	-1.12689
H	4.488644	2.710903	-0.32261
H	3.718696	2.611953	-1.91908
H	-1.41882	0.933147	-0.84715
H	-3.38917	-1.60147	1.987238
H	-5.54238	-1.09957	0.898415
H	-2.58765	2.149548	-2.4333
H	-4.03751	1.345952	-3.07087
H	-4.19265	2.686949	-1.93805
H	-6.28754	1.581326	-1.21832
H	-6.04182	0.193168	-2.27054
H	-6.8578	-0.01629	-0.71747
O	-2.25063	2.343745	0.682697
H	-3.0848	2.244924	1.165581

BF-C3'-OH-TS-G

Atom	X	Y	Z		
				Electronic Energy (EE)	-1158.66
O	0.608407	-2.32958	0.673842	Zero-point Energy Correction	0.496815
O	-1.31144	-3.09305	1.54341	Thermal Correction to Energy	0.524669
C	3.310409	-2.02056	-0.58113	Thermal Correction to Enthalpy	0.525613
C	2.406423	-0.86577	-0.15124	Thermal Correction to Free Energy	0.438774
C	2.318143	3.016989	-0.15331		
C	0.321821	-0.05604	0.835029		

C	1.926571	1.553119	0.069689
C	1.164175	-1.07996	0.430632
C	-0.92302	-0.67025	1.402261
C	2.746805	0.486397	-0.31222
C	0.691192	1.262929	0.659731
C	4.624558	-1.51744	-1.18653
C	2.585189	-2.87009	-1.63996
C	3.645401	-2.89021	0.643609
C	-0.6287	-2.16958	1.241558
C	2.327312	3.755975	1.195499
C	1.288243	3.67363	-1.08937
C	3.705168	3.160162	-0.78731
C	-2.23919	-0.29318	0.747174
C	-2.28964	0.36843	-0.46491
C	-3.44013	-0.64693	1.375268
C	-3.52104	0.759089	-1.0521
C	-4.73273	0.34664	-0.42031
C	-4.66275	-0.33139	0.789492
C	-3.52927	1.22859	-2.48247
C	-6.04988	0.709016	-1.03591
H	-1.00379	-0.48958	2.479839
H	3.701461	0.710351	-0.76487
H	0.012628	2.055033	0.958496
H	5.235801	-2.37619	-1.47314
H	4.453832	-0.91632	-2.0834
H	5.198907	-0.92339	-0.47088
H	2.341422	-2.26377	-2.5162
H	3.233942	-3.68969	-1.96122
H	1.663679	-3.29971	-1.24651
H	4.302036	-3.71132	0.343002
H	4.163505	-2.29908	1.403196
H	2.747028	-3.31865	1.088502
H	2.608441	4.802336	1.047905
H	1.3451	3.738156	1.672113
H	3.045581	3.299265	1.880996
H	0.279288	3.638833	-0.67212
H	1.54738	4.722842	-1.25667
H	1.271001	3.165785	-2.0572
H	3.934351	4.220215	-0.91881
H	4.484466	2.726333	-0.15524
H	3.750225	2.686417	-1.77129
H	-1.37415	0.648069	-0.97426
H	-3.41204	-1.18517	2.316347
H	-5.58155	-0.62297	1.286189
H	-2.57415	1.69201	-2.72853
H	-3.6938	0.381649	-3.15531

H	-4.3152	1.964506	-2.64586
H	-6.12918	1.797442	-1.11516
H	-6.14235	0.298475	-2.04512
H	-6.87986	0.338108	-0.43486
O	-3.54545	2.662898	-0.32692
H	-3.53037	2.485754	0.625555

BF-C3-Al-TS-B

Atom	X	Y	Z		
O	0.649516	-2.45227	0.482454	Electronic Energy (EE)	-1293.63
O	-1.34928	-3.2678	1.082871	Zero-point Energy Correction	0.571028
C	3.533909	-2.07392	-0.23705	Thermal Correction to Energy	0.604132
C	2.505155	-0.95059	-0.10139	Thermal Correction to Enthalpy	0.605076
C	2.292658	2.928059	-0.20925	Thermal Correction to Free Energy	0.506857
C	0.217034	-0.20185	0.361193		
C	1.899691	1.451917	-0.08994		
C	1.178731	-1.20006	0.216338		
C	-1.01713	-0.85074	0.847998		
C	2.819829	0.41047	-0.25554		
C	0.575919	1.130929	0.229643		
C	4.917166	-1.53485	-0.61363		
C	3.084497	-3.05452	-1.3334		
C	3.6563	-2.81606	1.105354		
C	-0.67319	-2.31697	0.827479		
C	2.041035	3.620595	1.141097		
C	1.433861	3.600796	-1.29273		
C	3.766634	3.112113	-0.58078		
C	-2.39597	-0.4548	0.452517		
C	-2.62199	0.539881	-0.50234		
C	-3.49611	-1.07559	1.050114		
C	-3.91005	0.924102	-0.86621		
C	-5.01218	0.300393	-0.258		
C	-4.78248	-0.69588	0.691171		
C	-4.11666	2.000621	-1.89796		
C	-6.41449	0.693307	-0.63483		
H	-0.95516	-0.59803	2.101978		
H	3.840456	0.662693	-0.50394		
H	-0.15825	1.908624	0.404075		
H	5.611447	-2.37301	-0.71131		
H	4.900352	-1.00447	-1.56954		
H	5.315339	-0.86266	0.151338		
H	2.998732	-2.54352	-2.29625		
H	3.822394	-3.85521	-1.4377		
H	2.122096	-3.5106	-1.09807		
H	4.41412	-3.60093	1.025935		

H	3.960842	-2.12898	1.900188
H	2.714521	-3.28502	1.393406
H	2.318215	4.677154	1.078891
H	0.99174	3.564736	1.440099
H	2.638575	3.156744	1.931239
H	0.369346	3.553096	-1.05107
H	1.706602	4.655979	-1.3859
H	1.584992	3.120802	-2.2636
H	3.988973	4.17941	-0.65737
H	4.432	2.690119	0.17741
H	4.003171	2.653469	-1.54497
H	-1.77911	1.012788	-0.99366
H	-3.34294	-1.85834	1.782995
H	-5.63113	-1.18424	1.15898
H	-3.16775	2.30275	-2.34254
H	-4.77692	1.661136	-2.70061
H	-4.57922	2.887174	-1.45338
H	-6.56614	1.771535	-0.53418
H	-6.62907	0.43503	-1.67655
H	-7.14539	0.18566	-0.00458
C	-0.69168	-0.19703	3.472618
C	-1.77842	-0.89479	4.250608
H	-2.76923	-0.54148	3.963072
H	-1.71899	-1.97352	4.08662
H	-1.6493	-0.71128	5.323918
C	0.722819	-0.69703	3.644829
H	0.764493	-1.77067	3.440167
H	1.418943	-0.17773	2.982971
H	1.053035	-0.54406	4.678687
C	-0.84063	1.215754	3.317117
N	-0.97026	2.347645	3.123244

BF-C3-CMO-TS-B

Atom	X	Y	Z		
O	0.53812	-2.47941	0.522143	Electronic Energy (EE)	-1507.62
O	-1.466	-3.32673	1.04849	Zero-point Energy Correction	0.657745
C	3.42478	-2.05591	-0.16926	Thermal Correction to Energy	0.694368
C	2.402688	-0.94257	0.060274	Thermal Correction to Enthalpy	0.695312
C	2.193947	2.936251	0.185761	Thermal Correction to Free Energy	0.587789
C	0.134635	-0.22383	0.632368		
C	1.803765	1.455588	0.24814		
C	1.079459	-1.21063	0.377402		
C	-1.11598	-0.89426	1.059211		
C	2.718605	0.42521	-0.00089		
C	0.488579	1.113124	0.580702		

C	4.809542	-1.49565	-0.50691
C	2.9675	-2.94649	-1.33692
C	3.545304	-2.89953	1.11235
C	-0.78047	-2.3657	0.891085
C	1.968117	3.576704	1.565712
C	1.314792	3.649518	-0.85494
C	3.66062	3.139247	-0.20459
C	-2.45766	-0.47009	0.545653
C	-2.58078	0.359496	-0.57055
C	-3.61219	-0.92093	1.183916
C	-3.82864	0.73861	-1.06073
C	-4.98912	0.279071	-0.41569
C	-4.85873	-0.54811	0.700124
C	-3.93177	1.628886	-2.27042
C	-6.34929	0.676589	-0.92107
H	-1.17551	-0.73023	2.243394
H	3.735537	0.69165	-0.24959
H	-0.25243	1.872034	0.807292
H	5.50052	-2.32693	-0.66695
H	4.793905	-0.89653	-1.42146
H	5.210742	-0.88319	0.305168
H	2.881861	-2.36242	-2.25735
H	3.70144	-3.74003	-1.50469
H	2.003668	-3.41519	-1.13478
H	4.299703	-3.67918	0.971784
H	3.852138	-2.27679	1.95763
H	2.601845	-3.38584	1.364599
H	2.245929	4.634463	1.53989
H	0.921907	3.513218	1.874051
H	2.577035	3.081333	2.3273
H	0.253977	3.583133	-0.60156
H	1.580065	4.709398	-0.90871
H	1.454648	3.211122	-1.84689
H	3.880314	4.209283	-0.23963
H	4.34075	2.685541	0.521358
H	3.878608	2.723371	-1.19209
H	-1.68791	0.708792	-1.079
H	-3.53035	-1.5538	2.060404
H	-5.75301	-0.90085	1.203205
H	-2.94293	1.898012	-2.64359
H	-4.47699	1.134587	-3.07983
H	-4.4707	2.552022	-2.03778
H	-6.47649	1.76313	-0.90388
H	-6.49695	0.353241	-1.95573
H	-7.13756	0.233397	-0.31169
O	-1.23192	-0.2929	3.573108

C	-0.43139	-1.06657	4.428614
C	0.954272	-1.32286	3.81515
C	-1.12464	-2.38851	4.74618
H	1.388927	-0.39845	3.428752
H	1.622642	-1.73688	4.573442
H	0.881465	-2.05081	3.002456
H	-2.09828	-2.21474	5.208165
H	-0.51824	-3.01505	5.404427
H	-1.28164	-2.93533	3.812753
C	-0.28097	-0.13993	5.649813
C	0.184228	1.163608	5.449084
C	-0.57339	-0.56326	6.944613
C	0.370664	2.01989	6.526084
H	0.388623	1.50475	4.440642
C	-0.37879	0.295109	8.025054
H	-0.94691	-1.56337	7.125131
C	0.092038	1.586223	7.820185
H	0.730526	3.028229	6.356242
H	-0.60119	-0.04982	9.02836
H	0.23733	2.253422	8.661841

BF-C3-CMO-TS-D

Atom	X	Y	Z		
O	0.534319	-2.49387	0.485681	Electronic Energy (EE)	-1507.61
O	-1.48361	-3.34352	0.938782	Zero-point Energy Correction	0.656533
C	3.44416	-2.05925	-0.12593	Thermal Correction to Energy	0.693171
C	2.409655	-0.95144	0.069473	Thermal Correction to Enthalpy	0.694115
C	2.183852	2.926996	0.176642	Thermal Correction to Free Energy	0.585831
C	0.128733	-0.238	0.597743		
C	1.798323	1.445759	0.238665		
C	1.080385	-1.22178	0.356455		
C	-1.12058	-0.91322	1.020771		
C	2.722624	0.41816	0.011379		
C	0.477996	1.100596	0.550566		
C	4.833485	-1.49172	-0.42885		
C	3.026966	-2.96036	-1.29971		
C	3.535223	-2.89143	1.165159		
C	-0.78724	-2.38036	0.827869		
C	1.934024	3.57081	1.550409		
C	1.315657	3.631973	-0.87818		
C	3.65479	3.133001	-0.19321		
C	-2.46832	-0.47888	0.534498		
C	-2.60697	0.365264	-0.56964		
C	-3.61399	-0.93038	1.19013		
C	-3.86275	0.757809	-1.03108		

C	-5.01474	0.296476	-0.36993
C	-4.8689	-0.54503	0.734445
C	-3.98449	1.665355	-2.22499
C	-6.3803	0.709457	-0.84504
H	-1.16234	-0.76046	2.207839
H	3.744039	0.687239	-0.21655
H	-0.26779	1.859656	0.761021
H	5.531773	-2.32158	-0.56352
H	4.837282	-0.89725	-1.34657
H	5.205902	-0.87014	0.390013
H	2.965655	-2.38213	-2.22605
H	3.772338	-3.74881	-1.43879
H	2.060403	-3.43483	-1.12374
H	4.298096	-3.66686	1.048559
H	3.817177	-2.25911	2.012395
H	2.588252	-3.38106	1.399125
H	2.208659	4.629403	1.521514
H	0.882705	3.50322	1.84102
H	2.534951	3.081564	2.322797
H	0.252017	3.560167	-0.63747
H	1.579039	4.692555	-0.92907
H	1.473469	3.192143	-1.86727
H	3.86795	4.204487	-0.22921
H	4.325314	2.683342	0.544343
H	3.887768	2.711658	-1.17515
H	-1.72292	0.719613	-1.09011
H	-3.52117	-1.57228	2.059813
H	-5.75641	-0.89736	1.250319
H	-3.00091	1.936471	-2.61042
H	-4.54879	1.183041	-3.02871
H	-4.51904	2.584251	-1.96647
H	-6.4967	1.796885	-0.80927
H	-6.54313	0.405938	-1.88354
H	-7.15973	0.259904	-0.22878
O	-1.19781	-0.29195	3.532274
C	-0.39277	-1.07007	4.382992
C	0.992336	-1.32166	3.769578
C	-1.08133	-2.39437	4.695429
H	1.427433	-0.39711	3.383326
H	1.659488	-1.73869	4.527553
H	0.915754	-2.05052	2.958143
H	-2.06512	-2.22916	5.138823
H	-0.48043	-3.00757	5.370896
H	-1.21202	-2.94675	3.760909
C	-0.25648	-0.14338	5.605973
C	0.270115	1.140155	5.42013

C	-0.63457	-0.54315	6.887148
C	0.431791	2.000071	6.499351
H	0.548937	1.465528	4.424065
C	-0.4647	0.318874	7.969951
H	-1.05235	-1.52759	7.057344
C	0.065842	1.590109	7.780473
H	0.841504	2.991401	6.341286
H	-0.75257	-0.00801	8.962858
H	0.191735	2.259708	8.623698

BF-C3-OH-TS-B

Atom	X	Y	Z		
O	0.632408	-2.31889	0.647793	Electronic Energy (EE)	-1158.68
O	-1.28296	-3.08053	1.519747	Zero-point Energy Correction	0.49155
C	3.368633	-2.00475	-0.54444	Thermal Correction to Energy	0.520042
C	2.441186	-0.8538	-0.15294	Thermal Correction to Enthalpy	0.520986
C	2.337309	3.031456	-0.18069	Thermal Correction to Free Energy	0.431581
C	0.320912	-0.04543	0.762947		
C	1.935787	1.567071	0.027552		
C	1.187022	-1.06886	0.399365		
C	-0.91855	-0.66265	1.305266		
C	2.773917	0.500408	-0.3201		
C	0.685165	1.276751	0.582352		
C	4.69208	-1.49513	-1.12264		
C	2.681413	-2.87611	-1.60989		
C	3.682315	-2.85441	0.699079		
C	-0.61162	-2.155	1.182789		
C	2.343945	3.754098	1.176541		
C	1.317277	3.707783	-1.11207		
C	3.727152	3.17349	-0.80768		
C	-2.25499	-0.26153	0.735288		
C	-2.34584	0.313363	-0.53414		
C	-3.42396	-0.4956	1.45454		
C	-3.57596	0.64904	-1.09496		
C	-4.75297	0.407386	-0.36618		
C	-4.65455	-0.16391	0.901625		
C	-3.64331	1.264881	-2.46687		
C	-6.09419	0.764553	-0.94635		
H	-0.96324	-0.5087	2.454421		
H	3.740185	0.726	-0.74696		
H	-0.0021	2.06595	0.867023		
H	5.318965	-2.35133	-1.38341		
H	4.540205	-0.90663	-2.03155		
H	5.244451	-0.88728	-0.40094		
H	2.450842	-2.28642	-2.50144		

H	3.347871	-3.69178	-1.90466
H	1.755312	-3.31558	-1.2372
H	4.357868	-3.67038	0.426805
H	4.171742	-2.2492	1.466994
H	2.780184	-3.29227	1.12813
H	2.62921	4.801638	1.042798
H	1.360506	3.733263	1.651588
H	3.058723	3.289086	1.860731
H	0.308589	3.68546	-0.69259
H	1.590072	4.755099	-1.271
H	1.290753	3.210008	-2.08544
H	3.958948	4.233936	-0.93443
H	4.50478	2.738365	-0.17414
H	3.77809	2.703265	-1.79357
H	-1.44088	0.498597	-1.10377
H	-3.37322	-0.92318	2.449062
H	-5.55978	-0.34675	1.471097
H	-2.64592	1.387934	-2.89052
H	-4.22887	0.643153	-3.15037
H	-4.12351	2.247285	-2.43596
H	-6.15708	1.833858	-1.16881
H	-6.27315	0.231205	-1.88458
H	-6.89916	0.513972	-0.25477
O	-1.11391	-0.91739	3.884383
H	-1.4001	-1.83881	3.749099

BF-C3-OH-TS-G

Atom	X	Y	Z		
O	0.634945	-2.31559	0.657195	Electronic Energy (EE)	-1158.66
O	-1.26488	-3.07323	1.575873	Zero-point Energy Correction	0.492394
C	3.35458	-1.99994	-0.55878	Thermal Correction to Energy	0.520629
C	2.434596	-0.84835	-0.15523	Thermal Correction to Enthalpy	0.521573
C	2.332934	3.035293	-0.172	Thermal Correction to Free Energy	0.433104
C	0.324953	-0.04218	0.784777		
C	1.933476	1.571344	0.037853		
C	1.186006	-1.06533	0.409006		
C	-0.9119	-0.65883	1.330152		
C	2.767127	0.505364	-0.32097		
C	0.688596	1.279664	0.603201		
C	4.673315	-1.49173	-1.1497		
C	2.652469	-2.86682	-1.61907		
C	3.677957	-2.85287	0.680776		
C	-0.60349	-2.15214	1.213766		
C	2.336607	3.760971	1.18409		
C	1.313554	3.706909	-1.10829		

C	3.724349	3.177812	-0.79661
C	-2.24447	-0.25797	0.752266
C	-2.32694	0.297863	-0.52592
C	-3.41679	-0.47253	1.471364
C	-3.55227	0.631884	-1.09642
C	-4.73242	0.409348	-0.36864
C	-4.64239	-0.14121	0.908195
C	-3.61002	1.225975	-2.47929
C	-6.0706	0.764822	-0.95921
H	-0.96604	-0.50597	2.484816
H	3.72909	0.732031	-0.75649
H	0.002146	2.066902	0.89498
H	5.295435	-2.34819	-1.41905
H	4.511149	-0.90046	-2.05474
H	5.232634	-0.88678	-0.43128
H	2.416904	-2.27244	-2.5057
H	3.312802	-3.68434	-1.92125
H	1.728771	-3.30027	-1.23505
H	4.346605	-3.67105	0.399431
H	4.178135	-2.24883	1.442057
H	2.776745	-3.2845	1.116682
H	2.621347	4.808062	1.048722
H	1.352249	3.739121	1.656117
H	3.049607	3.295284	1.868781
H	0.30428	3.678941	-0.69174
H	1.582593	4.754856	-1.2673
H	1.29383	3.205401	-2.07932
H	3.956526	4.237857	-0.92263
H	4.498674	2.741192	-0.16058
H	3.775318	2.706886	-1.78175
H	-1.41757	0.469076	-1.09315
H	-3.36772	-0.88125	2.473326
H	-5.55086	-0.30762	1.477198
H	-2.60986	1.338646	-2.89812
H	-4.19301	0.59489	-3.15588
H	-4.08726	2.209865	-2.4671
H	-6.12808	1.830925	-1.19639
H	-6.24837	0.219256	-1.89023
H	-6.87836	0.527048	-0.26708
O	-1.18521	-0.93838	3.873453
H	-1.41778	-1.86866	3.702816

BF-C3-OOH-TS-B

Atom	X	Y	Z		
				Electronic Energy (EE)	-1233.83
O	0.634366	-2.52615	0.340256	Zero-point Energy Correction	0.496216

O	-1.40256	-3.34794	0.79173	Thermal Correction to Energy	0.525677
C	3.530809	-2.13469	-0.3204	Thermal Correction to Enthalpy	0.526621
C	2.503688	-1.01397	-0.16041	Thermal Correction to Free Energy	0.435358
C	2.292954	2.867879	-0.21965		
C	0.216029	-0.27231	0.316592		
C	1.898702	1.391795	-0.09797		
C	1.17409	-1.27192	0.145344		
C	-1.02198	-0.92527	0.762699		
C	2.818583	0.348581	-0.2811		
C	0.57705	1.065687	0.217327		
C	4.917622	-1.58796	-0.67138		
C	3.087858	-3.083	-1.44772		
C	3.637896	-2.91208	1.003039		
C	-0.70227	-2.39714	0.644754		
C	2.031081	3.573281	1.121673		
C	1.442572	3.531278	-1.31578		
C	3.769939	3.051323	-0.5795		
C	-2.3935	-0.47202	0.407843		
C	-2.61108	0.456747	-0.61357		
C	-3.4922	-0.98488	1.098955		
C	-3.89398	0.878568	-0.95299		
C	-4.99616	0.361018	-0.251		
C	-4.77339	-0.56788	0.765694		
C	-4.09841	1.872216	-2.06504		
C	-6.39264	0.800225	-0.59623		
H	-0.92478	-0.78353	2.010891		
H	3.840028	0.60464	-0.52214		
H	-0.16622	1.836203	0.389865		
H	5.612107	-2.42443	-0.78017		
H	4.911397	-1.0371	-1.61603		
H	5.309057	-0.93374	0.112463		
H	3.00327	-2.54428	-2.39558		
H	3.830159	-3.87639	-1.57288		
H	2.127615	-3.55128	-1.22903		
H	4.380353	-3.70903	0.902863		
H	3.954791	-2.2516	1.815053		
H	2.68727	-3.3673	1.283151		
H	2.307354	4.6293	1.052188		
H	0.977624	3.523008	1.40712		
H	2.623622	3.11983	1.921631		
H	0.375739	3.482417	-1.08426		
H	1.714748	4.585891	-1.41583		
H	1.603147	3.043259	-2.2809		
H	3.993045	4.118441	-0.65478		
H	4.429857	2.628522	0.182906		
H	4.012838	2.593285	-1.54211		

H	-1.76619	0.84399	-1.17358
H	-3.3416	-1.70701	1.893359
H	-5.62208	-0.97076	1.308337
H	-3.14471	2.177499	-2.49709
H	-4.71315	1.448974	-2.86475
H	-4.61245	2.76843	-1.70564
H	-6.51249	1.879608	-0.46349
H	-6.62982	0.577607	-1.64072
H	-7.12724	0.297324	0.033308
H	1.163895	0.183264	2.881252
O	-0.5224	-0.527	3.228995
O	0.824148	-0.67945	3.175372

BF-C3-OOH-TS-D

Atom	X	Y	Z		
O	0.633462	-2.54111	0.352508	Electronic Energy (EE)	-1233.83
O	-1.39886	-3.36033	0.809724	Zero-point Energy Correction	0.495224
C	3.532269	-2.14486	-0.34135	Thermal Correction to Energy	0.524676
C	2.503353	-1.02796	-0.16889	Thermal Correction to Enthalpy	0.52562
C	2.293622	2.85369	-0.20305	Thermal Correction to Free Energy	0.434206
C	0.218558	-0.28588	0.320458		
C	1.897155	1.378053	-0.0994		
C	1.177026	-1.28416	0.146527		
C	-1.01717	-0.93661	0.773502		
C	2.816937	0.336197	-0.28981		
C	0.575465	1.052975	0.217807		
C	4.914503	-1.59211	-0.69899		
C	3.085119	-3.08773	-1.47079		
C	3.653616	-2.92918	0.976087		
C	-0.69752	-2.40639	0.658414		
C	2.043288	3.532385	1.153978		
C	1.434278	3.537114	-1.2789		
C	3.767471	3.038282	-0.57225		
C	-2.38904	-0.48419	0.414796		
C	-2.60121	0.438539	-0.6142		
C	-3.49146	-0.98644	1.109425		
C	-3.88301	0.861773	-0.95933		
C	-4.98956	0.352477	-0.25619		
C	-4.77221	-0.56867	0.769772		
C	-4.08252	1.850209	-2.07578		
C	-6.38237	0.794945	-0.6081		
H	-0.92084	-0.77577	2.019961		
H	3.83758	0.59307	-0.53462		
H	-0.16743	1.823489	0.393076		
H	5.608108	-2.42818	-0.81802		

H	4.897077	-1.03352	-1.63885
H	5.307309	-0.94048	0.086288
H	2.997779	-2.54333	-2.41534
H	3.828347	-3.87958	-1.60117
H	2.12489	-3.55728	-1.25187
H	4.402152	-3.71896	0.864278
H	3.971992	-2.2698	1.788747
H	2.708408	-3.39497	1.259398
H	2.327853	4.58741	1.101599
H	0.989864	3.481342	1.440415
H	2.636447	3.056123	1.940467
H	0.369351	3.481621	-1.03957
H	1.707485	4.593444	-1.3572
H	1.591082	3.069512	-2.25525
H	3.98878	4.106768	-0.6355
H	4.431571	2.603151	0.179694
H	4.000835	2.589591	-1.5419
H	-1.75518	0.825147	-1.17286
H	-3.34872	-1.69816	1.915037
H	-5.62392	-0.96196	1.315351
H	-3.12622	2.152054	-2.50457
H	-4.69836	1.422863	-2.87269
H	-4.59896	2.745951	-1.71841
H	-6.49462	1.875962	-0.48102
H	-6.61223	0.571639	-1.65422
H	-7.1202	0.296024	0.0211
H	1.021443	0.472435	2.865811
O	-0.56182	-0.44187	3.238311
O	0.792942	-0.41478	3.200474

BF-C3-OOH-TS-G

Atom	X	Y	Z		
				Electronic Energy (EE)	-1233.81
O	0.635489	-2.53197	0.325938	Zero-point Energy Correction	0.496972
O	-1.40424	-3.35045	0.795795	Thermal Correction to Energy	0.526259
C	3.52995	-2.14033	-0.31266	Thermal Correction to Enthalpy	0.527203
C	2.50267	-1.01912	-0.16788	Thermal Correction to Free Energy	0.436718
C	2.295036	2.861355	-0.21605		
C	0.211005	-0.2798	0.28667		
C	1.898534	1.385548	-0.10637		
C	1.170088	-1.27953	0.124021		
C	-1.02262	-0.92996	0.745125		
C	2.8194	0.342699	-0.28404		
C	0.573763	1.05791	0.193954		
C	4.920161	-1.59556	-0.65454		
C	3.094234	-3.09411	-1.43898		

C	3.623372	-2.90957	1.017431
C	-0.70515	-2.40355	0.638951
C	2.028353	3.557232	1.129961
C	1.452327	3.533486	-1.31352
C	3.774788	3.044916	-0.56668
C	-2.39507	-0.48041	0.396645
C	-2.61805	0.463798	-0.60845
C	-3.49008	-1.01242	1.078328
C	-3.90279	0.884638	-0.93969
C	-5.0007	0.350262	-0.24589
C	-4.7728	-0.59582	0.753015
C	-4.11347	1.895753	-2.036
C	-6.40077	0.788102	-0.58261
H	-0.91019	-0.75757	1.993499
H	3.843034	0.598553	-0.51462
H	-0.17156	1.82649	0.366797
H	5.614581	-2.43259	-0.75493
H	4.919697	-1.0477	-1.60073
H	5.303872	-0.93923	0.130996
H	3.020973	-2.55854	-2.38912
H	3.835402	-3.88993	-1.55193
H	2.13031	-3.55514	-1.22362
H	4.365271	-3.70789	0.928101
H	3.933777	-2.24241	1.825871
H	2.668113	-3.35865	1.289272
H	2.30056	4.614469	1.068795
H	0.975124	3.498764	1.413251
H	2.622192	3.098298	1.925315
H	0.384615	3.48396	-1.0886
H	1.727137	4.58779	-1.40562
H	1.618872	3.048426	-2.27851
H	3.999749	4.111669	-0.63596
H	4.427898	2.617171	0.19829
H	4.020511	2.589427	-1.52937
H	-1.77467	0.861114	-1.16363
H	-3.33201	-1.75144	1.855075
H	-5.61915	-1.01346	1.287654
H	-3.16241	2.208511	-2.4677
H	-4.73159	1.484478	-2.8389
H	-4.62626	2.78551	-1.66009
H	-6.52326	1.865715	-0.44131
H	-6.64106	0.572435	-1.62758
H	-7.13102	0.278111	0.045557
H	1.188764	0.263108	2.748413
O	-0.48594	-0.42527	3.176568
O	0.859222	-0.57726	3.109249

BF-C3-TB-TS-D

Atom	X	Y	Z		
O	0.55747	-2.45313	0.545439	Electronic Energy (EE)	-1315.9
O	-1.47048	-3.266	1.020303	Zero-point Energy Correction	0.602661
C	3.463676	-2.06818	-0.11484	Thermal Correction to Energy	0.636338
C	2.440851	-0.94456	0.049395	Thermal Correction to Enthalpy	0.637282
C	2.239205	2.935735	-0.03106	Thermal Correction to Free Energy	0.537055
C	0.173164	-0.19107	0.580777		
C	1.847378	1.46194	0.115026		
C	1.113992	-1.19191	0.366564		
C	-1.07637	-0.83931	1.043287		
C	2.762105	0.418615	-0.07572		
C	0.530226	1.141164	0.464291		
C	4.855066	-1.52433	-0.44991		
C	3.027393	-3.00642	-1.25205		
C	3.559506	-2.85538	1.20379		
C	-0.76214	-2.31431	0.886991		
C	2.001504	3.6577	1.305368		
C	1.366524	3.584152	-1.1181		
C	3.708476	3.113906	-0.42159		
C	-2.42758	-0.40565	0.566996		
C	-2.57829	0.390426	-0.57072		
C	-3.56505	-0.8126	1.264581		
C	-3.83831	0.779002	-1.02407		
C	-4.98217	0.363581	-0.31966		
C	-4.82419	-0.43035	0.817784		
C	-3.97301	1.634274	-2.25463		
C	-6.35202	0.773379	-0.78532		
H	-1.0975	-0.64925	2.231069		
H	3.781715	0.66953	-0.33087		
H	-0.20731	1.914941	0.648208		
H	5.545771	-2.36445	-0.55731		
H	4.856688	-0.96597	-1.38999		
H	5.23825	-0.8743	0.341442		
H	2.961972	-2.46066	-2.19761		
H	3.764445	-3.80604	-1.37027		
H	2.058424	-3.4655	-1.05011		
H	4.315768	-3.64004	1.107963		
H	3.853062	-2.19628	2.026308		
H	2.61113	-3.32957	1.462371		
H	2.280872	4.711527	1.214194		
H	0.951925	3.612589	1.606162		
H	2.605347	3.210649	2.100507		
H	0.304476	3.531184	-0.86554		

H	1.634207	4.638884	-1.23095
H	1.514683	3.088186	-2.0818
H	3.926728	4.180825	-0.51569
H	4.381916	2.700135	0.334178
H	3.932527	2.640178	-1.38148
H	-1.7006	0.709125	-1.12406
H	-3.46174	-1.41731	2.159567
H	-5.70533	-0.74752	1.366385
H	-2.99456	1.868057	-2.67592
H	-4.56657	1.127973	-3.02162
H	-4.4831	2.57453	-2.02504
H	-6.45234	1.862849	-0.80132
H	-6.54213	0.421094	-1.80363
H	-7.12381	0.366731	-0.13082
O	-1.10825	-0.1732	3.541666
C	-0.38714	-1.01461	4.396005
C	-0.28566	-0.18855	5.696212
C	1.016062	-1.28742	3.847188
C	-1.14883	-2.31355	4.64948
H	-1.28037	0.063565	6.067797
H	0.279229	0.728837	5.522369
H	0.231272	-0.79295	6.445536
H	1.497023	-0.35499	3.53955
H	1.631048	-1.7674	4.61224
H	0.967942	-1.9653	2.990476
H	-2.13432	-2.09886	5.069515
H	-0.60021	-2.95276	5.345736
H	-1.28062	-2.86244	3.71257

BF-C3-TBO-TS-B

Atom	X	Y	Z		
O	0.564871	-2.44186	0.590376	Electronic Energy (EE)	-1315.91
O	-1.44763	-3.25158	1.142148	Zero-point Energy Correction	0.603851
C	3.44147	-2.07081	-0.16876	Thermal Correction to Energy	0.637591
C	2.433259	-0.94026	0.037473	Thermal Correction to Enthalpy	0.638535
C	2.249581	2.941272	-0.01468	Thermal Correction to Free Energy	0.537868
C	0.181922	-0.18009	0.623512		
C	1.852924	1.467757	0.126841		
C	1.114896	-1.18413	0.391804		
C	-1.06845	-0.82369	1.09094		
C	2.756579	0.420984	-0.09186		
C	0.54337	1.150257	0.502834		
C	4.825346	-1.5359	-0.54852		
C	2.956606	-2.99699	-1.297		
C	3.5762	-2.871	1.138885		

C	-0.75088	-2.3028	0.96049
C	2.070014	3.645166	1.340733
C	1.34308	3.61298	-1.05963
C	3.704636	3.115887	-0.45866
C	-2.41395	-0.40288	0.584537
C	-2.54811	0.388702	-0.55757
C	-3.56194	-0.82161	1.255586
C	-3.80053	0.761958	-1.04084
C	-4.95439	0.335013	-0.36246
C	-4.81301	-0.45467	0.778928
C	-3.91536	1.611819	-2.27825
C	-6.31917	0.726219	-0.86046
H	-1.11053	-0.62251	2.27367
H	3.769682	0.668754	-0.37341
H	-0.1872	1.924591	0.709407
H	5.507417	-2.37844	-0.68634
H	4.800695	-0.9726	-1.48535
H	5.243219	-0.89546	0.233031
H	2.859544	-2.44403	-2.23534
H	3.680875	-3.80234	-1.45001
H	1.992555	-3.44972	-1.06208
H	4.320801	-3.66242	1.012548
H	3.903198	-2.2227	1.956926
H	2.632827	-3.33909	1.42372
H	2.348269	4.699884	1.257033
H	1.034984	3.597755	1.687037
H	2.702553	3.18353	2.103938
H	0.290551	3.568782	-0.76961
H	1.614692	4.666689	-1.1716
H	1.447526	3.127299	-2.03379
H	3.92858	4.181627	-0.55109
H	4.404389	2.692982	0.267255
H	3.890287	2.652078	-1.43137
H	-1.66062	0.712387	-1.09173
H	-3.47054	-1.42539	2.151554
H	-5.70214	-0.78282	1.307223
H	-2.93041	1.855738	-2.67811
H	-4.48128	1.097475	-3.06045
H	-4.43866	2.549125	-2.06748
H	-6.43686	1.813739	-0.88187
H	-6.48641	0.365896	-1.87983
H	-7.10123	0.313219	-0.22251
O	-1.1546	-0.17706	3.594602
C	-0.41883	-1.00088	4.451705
C	-0.34419	-0.16916	5.74992
C	0.993627	-1.23845	3.908278

C	-1.15055	-2.31845	4.703137
H	-1.34719	0.064856	6.110264
H	0.199223	0.760678	5.575461
H	0.178278	-0.75477	6.510325
H	1.450518	-0.29432	3.601274
H	1.620312	-1.70403	4.672914
H	0.969212	-1.91428	3.049047
H	-2.14196	-2.1252	5.119126
H	-0.59264	-2.94728	5.401712
H	-1.2703	-2.87228	3.768056

BF-C4'-OH-TS-G

Atom	X	Y	Z	Electronic Energy (EE)	-1158.66
O	0.574382	-2.27708	0.678978	Zero-point Energy Correction	0.496734
O	-1.32685	-3.01805	1.606502	Thermal Correction to Energy	0.524648
C	3.257199	-2.00638	-0.61938	Thermal Correction to Enthalpy	0.525592
C	2.381466	-0.83894	-0.16576	Thermal Correction to Free Energy	0.438285
C	2.383112	3.043572	-0.10283		
C	0.324885	-0.00019	0.856447		
C	1.954534	1.585829	0.092522		
C	1.143836	-1.03534	0.432096		
C	-0.92274	-0.59761	1.437229		
C	2.746638	0.50776	-0.31579		
C	0.719491	1.313028	0.691987		
C	4.568464	-1.52186	-1.24589		
C	2.498076	-2.83633	-1.66992		
C	3.599547	-2.88952	0.593669		
C	-0.64487	-2.10335	1.280509		
C	2.435664	3.747716	1.263576		
C	1.358529	3.753789	-1.00443		
C	3.762904	3.164182	-0.7571		
C	-2.2391	-0.23025	0.781363		
C	-2.28811	0.341153	-0.49123		
C	-3.43492	-0.52299	1.449297		
C	-3.49633	0.63825	-1.10782		
C	-4.71927	0.3975	-0.41256		
C	-4.645	-0.23741	0.853889		
C	-3.53194	1.268401	-2.46703		
C	-6.0335	0.430969	-1.14565		
H	-0.99968	-0.41043	2.513533		
H	3.699864	0.717803	-0.77788		
H	0.065127	2.115133	1.01625		
H	5.15803	-2.38902	-1.55178		
H	4.391495	-0.90966	-2.13405		
H	5.16773	-0.94513	-0.53653		

H	2.249116	-2.22041	-2.53799
H	3.126534	-3.66488	-2.00795
H	1.576597	-3.25268	-1.26244
H	4.23724	-3.71902	0.275923
H	4.140664	-2.31211	1.347719
H	2.702456	-3.30653	1.051731
H	2.74166	4.789915	1.137495
H	1.462132	3.742364	1.75816
H	3.153595	3.255038	1.923921
H	0.358466	3.743006	-0.56538
H	1.648394	4.797532	-1.15417
H	1.304446	3.268051	-1.98209
H	4.019176	4.220447	-0.86726
H	4.540131	2.69453	-0.14874
H	3.778786	2.712922	-1.75249
H	-1.3636	0.557681	-1.01586
H	-3.4029	-0.99514	2.424696
H	-5.57151	-0.46659	1.368275
H	-2.52628	1.399235	-2.86629
H	-4.1086	0.661765	-3.17058
H	-4.0167	2.246901	-2.39995
H	-6.11742	1.329484	-1.75529
H	-6.12396	-0.44524	-1.79479
H	-6.85828	0.426656	-0.43374
O	-4.92329	2.324927	0.227441
H	-4.22845	2.362772	0.901292

BF-C4-OH-TS-G

Atom	X	Y	Z		
O	0.609475	-2.35981	0.574823	Electronic Energy (EE)	-1158.66
O	-1.31427	-3.14214	1.421912	Zero-point Energy Correction	0.496459
C	3.341901	-2.01472	-0.60421	Thermal Correction to Energy	0.52463
C	2.430383	-0.87312	-0.16578	Thermal Correction to Enthalpy	0.525574
C	2.326731	3.006745	-0.1401	Thermal Correction to Free Energy	0.437348
C	0.324887	-0.10048	0.795624		
C	1.967188	1.543967	0.114033		
C	1.165756	-1.10631	0.375889		
C	-0.91144	-0.7144	1.364959		
C	2.780444	0.487608	-0.28421		
C	0.730901	1.241659	0.75463		
C	4.670039	-1.50116	-1.16882		
C	2.636148	-2.83819	-1.69745		
C	3.646323	-2.91337	0.608788		
C	-0.63245	-2.2097	1.155525		
C	2.268586	3.810535	1.169341		

C	1.303167	3.58489	-1.13646
C	3.725539	3.165729	-0.74377
C	-2.23555	-0.30142	0.753423
C	-2.30125	0.276931	-0.51277
C	-3.41649	-0.53608	1.449247
C	-3.51932	0.629889	-1.09023
C	-4.70853	0.393018	-0.3833
C	-4.6357	-0.19097	0.87984
C	-3.55859	1.257654	-2.45876
C	-6.03885	0.765318	-0.98221
H	-0.94059	-0.54178	2.447436
H	3.736361	0.717547	-0.73056
H	-0.00121	2.019712	0.928739
H	5.285969	-2.35521	-1.45886
H	4.521242	-0.88205	-2.05728
H	5.229419	-0.92338	-0.42844
H	2.412184	-2.21189	-2.56483
H	3.292143	-3.65014	-2.02282
H	1.706632	-3.27561	-1.33322
H	4.305493	-3.7299	0.301877
H	4.152789	-2.343	1.391858
H	2.736937	-3.34718	1.024693
H	2.484582	4.861669	0.958889
H	1.291787	3.743896	1.649143
H	3.004722	3.439758	1.885805
H	0.28724	3.548992	-0.73667
H	1.542304	4.630196	-1.35038
H	1.320906	3.028119	-2.07708
H	3.935424	4.2278	-0.88899
H	4.496344	2.761071	-0.0826
H	3.807353	2.675972	-1.71755
H	-1.38497	0.460135	-1.06653
H	-3.38285	-0.99537	2.430866
H	-5.5521	-0.37702	1.429828
H	-2.55314	1.377745	-2.86286
H	-4.13493	0.644845	-3.15753
H	-4.03357	2.242285	-2.42868
H	-6.09262	1.837216	-1.19269
H	-6.20595	0.243633	-1.92874
H	-6.85566	0.511242	-0.30659
O	1.176475	1.34041	2.724202
H	2.018939	0.861735	2.697525

BF-C5'-OH-TS-G

Atom	X	Y	Z	Electronic Energy (EE)	-1158.66

O	0.596307	-2.29978	0.635065	Zero-point Energy Correction	0.496455
O	-1.29465	-3.08256	1.550143	Thermal Correction to Energy	0.524704
C	3.254966	-1.96592	-0.70037	Thermal Correction to Enthalpy	0.525648
C	2.381276	-0.82132	-0.1885	Thermal Correction to Free Energy	0.436525
C	2.361003	3.056601	0.009566		
C	0.337881	-0.03223	0.896935		
C	1.941667	1.59054	0.157211		
C	1.156298	-1.04735	0.425151		
C	-0.90161	-0.65652	1.470772		
C	2.734908	0.532456	-0.29858		
C	0.717175	1.288767	0.764822		
C	4.552549	-1.45105	-1.33115		
C	2.481282	-2.75997	-1.76789		
C	3.622895	-2.89236	0.472141		
C	-0.61992	-2.15258	1.255201		
C	2.440333	3.70613	1.401599		
C	1.316141	3.798902	-0.84119		
C	3.726303	3.20855	-0.6682		
C	-2.21265	-0.25645	0.824949		
C	-2.27086	0.021021	-0.55068		
C	-3.37459	-0.21977	1.565639		
C	-3.47125	0.319533	-1.1931		
C	-4.65445	0.355957	-0.44668		
C	-4.59576	0.148078	0.953327		
C	-3.49601	0.595153	-2.67372		
C	-5.96605	0.697889	-1.0872		
H	-0.97403	-0.50468	2.551926		
H	3.679223	0.764836	-0.76815		
H	0.061736	2.074175	1.125702		
H	5.14115	-2.3028	-1.67932		
H	4.357058	-0.8072	-2.19267		
H	5.161101	-0.89762	-0.61121		
H	2.215041	-2.11326	-2.60813		
H	3.107035	-3.5724	-2.14756		
H	1.568917	-3.19565	-1.36001		
H	4.258792	-3.70593	0.112315		
H	4.174368	-2.3408	1.237948		
H	2.735869	-3.33091	0.929573		
H	2.739646	4.753979	1.310896		
H	1.477764	3.67595	1.916386		
H	3.174192	3.190661	2.02603		
H	0.326214	3.768425	-0.38098		
H	1.601175	4.84842	-0.9558		
H	1.241134	3.351558	-1.83557		
H	3.975497	4.269491	-0.74462		
H	4.518157	2.72119	-0.09361		

H	3.723589	2.793924	-1.67955
H	-1.35337	-0.00019	-1.132
H	-3.36042	-0.43987	2.62665
H	-5.51915	-0.01211	1.49407
H	-2.49077	0.560982	-3.09376
H	-4.10888	-0.14126	-3.20045
H	-3.92236	1.579575	-2.88462
H	-5.9488	1.723776	-1.46699
H	-6.17937	0.038405	-1.9329
H	-6.78055	0.62579	-0.36785
O	-4.76989	2.073805	1.48059
H	-3.91029	2.380963	1.155726

BF-C5-OH-TS-G

Atom	X	Y	Z		
O	0.588706	-2.33816	0.608933	Electronic Energy (EE)	-1158.66
O	-1.30974	-3.14414	1.490792	Zero-point Energy Correction	0.496119
C	3.258124	-2.00423	-0.69504	Thermal Correction to Energy	0.524366
C	2.398315	-0.86439	-0.1503	Thermal Correction to Enthalpy	0.52531
C	2.299422	2.993091	-0.09597	Thermal Correction to Free Energy	0.436973
C	0.342882	-0.08412	0.958333		
C	2.004451	1.549354	0.335177		
C	1.157704	-1.09438	0.450993		
C	-0.9098	-0.71584	1.490747		
C	2.768866	0.475541	-0.2067		
C	0.736162	1.229735	0.889785		
C	4.561644	-1.48154	-1.30647		
C	2.479658	-2.75951	-1.78646		
C	3.614195	-2.96548	0.452893		
C	-0.63772	-2.20388	1.231447		
C	1.779824	4.007763	0.931788		
C	1.567798	3.21783	-1.4327		
C	3.800551	3.23437	-0.30239		
C	-2.21744	-0.28861	0.847598		
C	-2.25066	0.296903	-0.41653		
C	-3.41563	-0.52859	1.511629		
C	-3.45447	0.650889	-1.02256		
C	-4.66139	0.410375	-0.34719		
C	-4.62052	-0.17998	0.914273		
C	-3.45944	1.285685	-2.3883		
C	-5.97603	0.785698	-0.97766		
H	-0.99292	-0.59914	2.575988		
H	3.719195	0.725835	-0.65431		
H	0.091531	2.009923	1.273955		
H	5.143152	-2.32723	-1.68009		

H	4.37312	-0.80972	-2.14806
H	5.173164	-0.95567	-0.56872
H	2.220619	-2.08632	-2.60782
H	3.100194	-3.56492	-2.1886
H	1.562897	-3.20075	-1.39507
H	4.24325	-3.77495	0.072812
H	4.170998	-2.44046	1.233769
H	2.722409	-3.40893	0.896549
H	2.05337	5.015113	0.608539
H	0.691217	3.987117	1.017243
H	2.214918	3.819659	1.913233
H	0.487856	3.089011	-1.31552
H	1.753864	4.233294	-1.79363
H	1.915603	2.513744	-2.19362
H	3.967111	4.293946	-0.5103
H	4.359864	2.958137	0.592139
H	4.193174	2.678254	-1.15697
H	-1.32121	0.487566	-0.94402
H	-3.40776	-0.99576	2.490261
H	-5.55026	-0.36901	1.440254
H	-2.44449	1.405257	-2.76784
H	-4.02071	0.677954	-3.10351
H	-3.93267	2.271309	-2.36419
H	-6.02328	1.858213	-1.18632
H	-6.11963	0.267005	-1.92963
H	-6.80972	0.530363	-0.32352
O	3.040111	1.615578	2.125035
H	2.973551	0.679348	2.36372

BF-C6'-OH-TS-G

Atom	X	Y	Z		
O	0.616829	-2.30895	0.680507	Electronic Energy (EE)	-1158.66
O	-1.2906	-3.0659	1.581826	Zero-point Energy Correction	0.496603
C	3.315381	-2.01337	-0.58372	Thermal Correction to Energy	0.524743
C	2.413057	-0.85424	-0.16223	Thermal Correction to Enthalpy	0.525687
C	2.354739	3.028449	-0.14432	Thermal Correction to Free Energy	0.437699
C	0.328061	-0.03491	0.816076		
C	1.943918	1.566554	0.057758		
C	1.169914	-1.06262	0.419781		
C	-0.91353	-0.64284	1.394286		
C	2.758415	0.496304	-0.32635		
C	0.704926	1.282132	0.643858		
C	4.627571	-1.51682	-1.19872		
C	2.586614	-2.87366	-1.63133		
C	3.654176	-2.87066	0.648714		

C	-0.61474	-2.14343	1.261986
C	2.390373	3.740726	1.218407
C	1.326064	3.720778	-1.05513
C	3.736004	3.163626	-0.79257
C	-2.22757	-0.30009	0.732373
C	-2.31018	0.331541	-0.49665
C	-3.41653	-0.60374	1.436753
C	-3.54522	0.641958	-1.07572
C	-4.73928	0.30311	-0.40199
C	-4.65832	-0.33556	0.823371
C	-3.59989	1.326586	-2.41286
C	-6.07579	0.617144	-1.02041
H	-1.02249	-0.41958	2.461835
H	3.714833	0.715502	-0.77757
H	0.034433	2.074549	0.959187
H	5.236133	-2.37858	-1.48203
H	4.453573	-0.92153	-2.09892
H	5.206358	-0.91887	-0.48994
H	2.340699	-2.27655	-2.51335
H	3.23379	-3.69717	-1.94573
H	1.666007	-3.29831	-1.23043
H	4.310425	-3.69443	0.354517
H	4.173824	-2.27167	1.401002
H	2.757152	-3.29475	1.100375
H	2.681684	4.786787	1.088518
H	1.415042	3.721997	1.709018
H	3.112344	3.261828	1.884367
H	0.324629	3.69985	-0.61946
H	1.603559	4.767265	-1.2097
H	1.282784	3.228418	-2.03013
H	3.977618	4.22251	-0.91109
H	4.516611	2.711182	-0.17565
H	3.763968	2.702833	-1.78338
H	-1.40017	0.596	-1.02494
H	-3.37196	-1.27104	2.287105
H	-5.56653	-0.61273	1.346856
H	-2.59815	1.517007	-2.79738
H	-4.13747	0.715798	-3.14394
H	-4.12921	2.281577	-2.34604
H	-6.19137	1.690958	-1.19158
H	-6.18905	0.122694	-1.989
H	-6.88912	0.287448	-0.37426
O	-3.20932	0.910649	2.780595
H	-3.53805	1.628822	2.219729

BF-C6-OH-TS-B

Atom	X	Y	Z		
O	0.564814	-2.32825	0.55768	Zero-point Energy Correction	0.496205
O	-1.34405	-3.13137	1.402256	Thermal Correction to Energy	0.524307
C	3.276095	-1.96131	-0.69023	Thermal Correction to Enthalpy	0.525252
C	2.362166	-0.84219	-0.201	Thermal Correction to Free Energy	0.437091
C	2.300976	3.055219	0.030357		
C	0.29364	-0.0641	0.84666		
C	1.883891	1.591019	0.16237		
C	1.132411	-1.07516	0.376275		
C	-0.94992	-0.70318	1.394163		
C	2.746573	0.531819	-0.23897		
C	0.655362	1.267495	0.735453		
C	4.566921	-1.40921	-1.30298		
C	2.547804	-2.79894	-1.75542		
C	3.653163	-2.8515	0.507468		
C	-0.66512	-2.19002	1.146767		
C	2.390539	3.676079	1.435346		
C	1.245643	3.809184	-0.7967		
C	3.662008	3.215641	-0.65331		
C	-2.25479	-0.26695	0.758048		
C	-2.34028	-0.09003	-0.62393		
C	-3.38775	-0.06477	1.535603		
C	-3.53227	0.281726	-1.23797		
C	-4.67727	0.488592	-0.44615		
C	-4.58453	0.31076	0.931483		
C	-3.59831	0.459736	-2.73082		
C	-5.97813	0.895982	-1.08324		
H	-1.02054	-0.57624	2.479381		
H	3.603169	0.78389	-0.84297		
H	-0.02252	2.039401	1.082755		
H	5.199122	-2.24584	-1.61056		
H	4.367453	-0.80387	-2.19203		
H	5.123997	-0.80819	-0.58066		
H	2.262951	-2.17796	-2.6094		
H	3.21463	-3.5866	-2.11764		
H	1.650342	-3.27312	-1.35667		
H	4.312883	-3.65718	0.1726		
H	4.18273	-2.26427	1.261687		
H	2.772325	-3.30479	0.965307		
H	2.681384	4.727821	1.359173		
H	1.432239	3.630869	1.958675		
H	3.139394	3.151522	2.03325		
H	0.259735	3.778663	-0.32748		
H	1.535953	4.858815	-0.8982		
H	1.156548	3.3815	-1.79911		

Note: the header row's last two cells read "Electronic Energy (EE)" and "-1158.68".

H	3.916682	4.277463	-0.69981
H	4.447341	2.70078	-0.09564
H	3.647188	2.836646	-1.67936
H	-1.45695	-0.24277	-1.23869
H	-3.34117	-0.20395	2.610359
H	-5.46492	0.468756	1.545756
H	-2.62726	0.273959	-3.19079
H	-4.3228	-0.22754	-3.17733
H	-3.9122	1.473646	-2.99529
H	-5.87908	1.848427	-1.6124
H	-6.30838	0.154717	-1.81668
H	-6.76316	1.004428	-0.33417
O	4.011776	0.598156	1.349589
H	3.371064	0.344365	2.031636

BF-C6-OH-TS-G

Atom	X	Y	Z		
O	0.562524	-2.31389	0.645073	Electronic Energy (EE)	-1158.66
O	-1.34235	-3.0745	1.550174	Zero-point Energy Correction	0.496793
C	3.26596	-2.00053	-0.62313	Thermal Correction to Energy	0.524846
C	2.355639	-0.86093	-0.17921	Thermal Correction to Enthalpy	0.525791
C	2.320616	3.04141	-0.06839	Thermal Correction to Free Energy	0.437884
C	0.292507	-0.03901	0.843277		
C	1.890262	1.585463	0.101354		
C	1.126683	-1.06989	0.410348		
C	-0.95154	-0.65153	1.412208		
C	2.744161	0.508715	-0.27094		
C	0.663265	1.286271	0.688507		
C	4.554841	-1.47619	-1.26478		
C	2.530224	-2.88459	-1.64559		
C	3.647452	-2.83468	0.614016		
C	-0.66563	-2.1517	1.239615		
C	2.443963	3.685569	1.324298		
C	1.259905	3.792911	-0.89144		
C	3.672446	3.170713	-0.77775		
C	-2.26364	-0.26438	0.755925		
C	-2.30791	0.197368	-0.55842		
C	-3.45398	-0.41734	1.458071		
C	-3.51512	0.512543	-1.17803		
C	-4.71468	0.360328	-0.46408		
C	-4.6628	-0.10569	0.847762		
C	-3.53271	1.011206	-2.59892		
C	-6.03333	0.696385	-1.1082		
H	-1.02933	-0.47372	2.490115		
H	3.595763	0.735182	-0.89165		

H	-0.01025	2.071856	1.012431
H	5.184498	-2.32619	-1.53755
H	4.349954	-0.91179	-2.17909
H	5.112394	-0.84408	-0.57028
H	2.244467	-2.30091	-2.5247
H	3.194478	-3.68887	-1.97327
H	1.633815	-3.33526	-1.21946
H	4.302147	-3.65684	0.312086
H	4.184246	-2.21152	1.333006
H	2.766201	-3.26094	1.095459
H	2.741187	4.733138	1.223809
H	1.495493	3.655123	1.865767
H	3.200346	3.161408	1.911859
H	0.28388	3.787887	-0.40156
H	1.563815	4.83506	-1.02161
H	1.146042	3.343014	-1.88117
H	3.937589	4.227999	-0.85219
H	4.458365	2.658374	-0.21927
H	3.634252	2.768747	-1.79424
H	-1.38366	0.319471	-1.11499
H	-3.43767	-0.78931	2.476586
H	-5.58681	-0.22654	1.403119
H	-2.52248	1.074373	-3.00355
H	-4.11608	0.346935	-3.24256
H	-3.98883	2.003009	-2.66416
H	-6.06684	1.744403	-1.41901
H	-6.20139	0.090751	-2.0031
H	-6.85996	0.520152	-0.4199
O	4.024182	0.629953	1.29007
H	3.402932	0.382896	1.991775

BF-C7-OH-TS-G

Atom	X	Y	Z		
O	0.638006	-2.25687	0.873412	Electronic Energy (EE)	-1158.66
O	-1.29238	-3.01569	1.729906	Zero-point Energy Correction	0.496362
C	3.254698	-1.95327	-0.59748	Thermal Correction to Energy	0.524496
C	2.478566	-0.82293	0.092855	Thermal Correction to Enthalpy	0.52544
C	2.298709	3.068393	-0.14348	Thermal Correction to Free Energy	0.437278
C	0.33466	0.008698	0.977773		
C	1.930567	1.608723	0.136352		
C	1.187691	-1.02479	0.626479		
C	-0.91829	-0.59065	1.545796		
C	2.764549	0.550718	-0.18955		
C	0.6895	1.32154	0.741699		
C	4.656441	-1.48561	-1.00612		

C	2.456394	-2.32615	-1.86119
C	3.414805	-3.20175	0.284631
C	-0.61731	-2.09168	1.425448
C	2.32638	3.847116	1.182668
C	1.243904	3.685483	-1.07778
C	3.670882	3.204026	-0.80985
C	-2.21974	-0.23559	0.854039
C	-2.24577	0.129605	-0.49127
C	-3.4187	-0.31983	1.552502
C	-3.44216	0.414015	-1.14512
C	-4.65064	0.331129	-0.43432
C	-4.61756	-0.03741	0.908522
C	-3.43963	0.806115	-2.59916
C	-5.95833	0.635991	-1.11526
H	-1.01345	-0.38219	2.616531
H	3.723144	0.759677	-0.63951
H	-0.00184	2.115995	1.001265
H	5.21607	-2.34116	-1.3904
H	4.630113	-0.73351	-1.79858
H	5.191954	-1.08386	-0.14291
H	2.313452	-1.45487	-2.50659
H	2.999662	-3.08721	-2.4276
H	1.476275	-2.73281	-1.60019
H	3.913158	-3.97604	-0.30514
H	4.025187	-2.97836	1.158097
H	2.458213	-3.59625	0.620675
H	2.586485	4.893156	0.999106
H	1.356322	3.826412	1.68411
H	3.06972	3.423712	1.863257
H	0.245491	3.659799	-0.63566
H	1.491445	4.730062	-1.28557
H	1.207967	3.145026	-2.0271
H	3.882641	4.260635	-0.98905
H	4.468258	2.806938	-0.1765
H	3.703209	2.689662	-1.77378
H	-1.31424	0.198321	-1.04514
H	-3.41757	-0.61498	2.596046
H	-5.54829	-0.10466	1.461743
H	-2.42527	0.821422	-2.99816
H	-4.02979	0.105993	-3.19696
H	-3.87743	1.798057	-2.74219
H	-5.97271	1.65702	-1.50686
H	-6.12834	-0.03479	-1.96207
H	-6.79342	0.526084	-0.42337
O	3.441299	-0.85855	1.896344
H	3.197855	0.026185	2.208024

BF-C8-OH-TS-G

Atom	X	Y	Z		
O	0.636776	-2.34646	0.506834	Electronic Energy (EE)	-1158.66
O	-1.33847	-3.1587	1.190001	Zero-point Energy Correction	0.496258
C	3.392159	-1.98047	-0.60147	Thermal Correction to Energy	0.524376
C	2.480779	-0.85164	-0.13634	Thermal Correction to Enthalpy	0.52532
C	2.287292	3.018251	-0.12877	Thermal Correction to Free Energy	0.437118
C	0.320062	-0.08501	0.791461		
C	1.916889	1.548068	0.071396		
C	1.240285	-1.11369	0.489908		
C	-0.92029	-0.73887	1.315821		
C	2.786623	0.501199	-0.27935		
C	0.653848	1.231783	0.6035		
C	4.730234	-1.44687	-1.12298		
C	2.695413	-2.73961	-1.74787		
C	3.674257	-2.9458	0.563481		
C	-0.63337	-2.2211	1.029046		
C	2.24173	3.739836	1.229801		
C	1.271591	3.66695	-1.08629		
C	3.688531	3.197193	-0.72125		
C	-2.24098	-0.28465	0.738596		
C	-2.34105	0.122562	-0.59197		
C	-3.38869	-0.30815	1.521554		
C	-3.5581	0.506817	-1.1474		
C	-4.7148	0.484048	-0.34987		
C	-4.60955	0.073639	0.976365		
C	-3.63516	0.93985	-2.58781		
C	-6.04456	0.894609	-0.9244		
H	-0.94064	-0.65656	2.408213		
H	3.744481	0.755362	-0.70757		
H	-0.06219	2.007181	0.851182		
H	5.35475	-2.29118	-1.42281		
H	4.6014	-0.80368	-1.99761		
H	5.269943	-0.88974	-0.35262		
H	2.480164	-2.06654	-2.58217		
H	3.35576	-3.53222	-2.11022		
H	1.763448	-3.19732	-1.41683		
H	4.308364	-3.76076	0.203387		
H	4.190979	-2.43045	1.375374		
H	2.756609	-3.36752	0.971194		
H	2.503177	4.793713	1.101508		
H	1.248041	3.691911	1.679474		
H	2.953004	3.292588	1.928854		
H	0.254446	3.618112	-0.6925		

H	1.523309	4.72005	-1.23834
H	1.284085	3.166472	-2.05773
H	3.897673	4.263259	-0.83519
H	4.458985	2.774601	-0.07133
H	3.77235	2.734971	-1.70804
H	-1.44933	0.143964	-1.21202
H	-3.3314	-0.63281	2.554621
H	-5.49994	0.051987	1.595733
H	-2.65267	0.906338	-3.05895
H	-4.3076	0.29307	-3.15826
H	-4.0206	1.959617	-2.67469
H	-6.01888	1.926364	-1.28629
H	-6.31902	0.26331	-1.77416
H	-6.83343	0.817079	-0.17615
O	1.831572	-1.22982	2.438565
H	2.289134	-0.38774	2.580234

BF-C9-OH-TS-G

Atom	X	Y	Z		
O	0.549819	-2.25014	0.880517	Electronic Energy (EE)	-1158.66
O	-1.2173	-3.01717	2.035082	Zero-point Energy Correction	0.496374
C	3.022311	-1.9486	-0.76907	Thermal Correction to Energy	0.524557
C	2.231138	-0.80079	-0.14318	Thermal Correction to Enthalpy	0.525501
C	2.236368	3.088248	0.017552	Thermal Correction to Free Energy	0.437395
C	0.382638	0.003272	1.274997		
C	1.838489	1.626482	0.250652		
C	1.101612	-1.02629	0.630831		
C	-0.91003	-0.59117	1.760854		
C	2.569286	0.551533	-0.29664		
C	0.717787	1.340531	1.015944		
C	4.230433	-1.43809	-1.56036		
C	2.112937	-2.73494	-1.72999		
C	3.530369	-2.87768	0.347521		
C	-0.60656	-2.08682	1.634554		
C	2.528536	3.754513	1.372545		
C	1.075373	3.822543	-0.67475		
C	3.48221	3.223104	-0.86378		
C	-2.12677	-0.24555	0.919733		
C	-2.01754	0.047803	-0.43992		
C	-3.38883	-0.26748	1.50281		
C	-3.13892	0.324822	-1.2179		
C	-4.41077	0.305941	-0.62242		
C	-4.51386	0.007304	0.734023		
C	-2.98863	0.642056	-2.68245		
C	-5.63979	0.603504	-1.43964		

H	-1.09657	-0.37295	2.811652
H	3.443406	0.781336	-0.88809
H	0.107961	2.124928	1.449021
H	4.766145	-2.29184	-1.98116
H	3.929593	-0.79293	-2.39022
H	4.929112	-0.88816	-0.92421
H	1.741082	-2.08361	-2.52555
H	2.681712	-3.54661	-2.19177
H	1.260374	-3.17074	-1.20915
H	4.099229	-3.70261	-0.09004
H	4.189324	-2.33301	1.029025
H	2.707065	-3.29846	0.925282
H	2.809563	4.800869	1.224545
H	1.657083	3.728525	2.029869
H	3.350886	3.246885	1.883232
H	0.16724	3.803849	-0.06887
H	1.342869	4.86855	-0.84792
H	0.847685	3.36093	-1.63907
H	3.714876	4.281851	-0.9987
H	4.354464	2.747755	-0.40751
H	3.325934	2.788619	-1.85466
H	-1.03803	0.068236	-0.91017
H	-3.49246	-0.50524	2.555681
H	-5.49387	-0.00926	1.199062
H	-1.94058	0.61784	-2.98167
H	-3.5355	-0.07613	-3.29976
H	-3.38693	1.633718	-2.91496
H	-5.59199	1.60446	-1.87778
H	-5.74495	-0.10416	-2.26676
H	-6.53938	0.544205	-0.82691
O	1.263263	-0.32443	3.080126
H	2.180027	-0.08048	2.883276

Table S5: Cartesian coordinates and electronic energies of all optimized species involved in the SET reactions between BF and HO•/HOO•/AI•/CMO•/TBO•/DPPH in the DMSO

Cartesian Coordinates				Energy	
AI-ANION-V					
Atom	X	Y	Z	Electronic Energy (EE)	-210.819
C	-0.64658	-0.48303	0.414009		
C	0.020921	0.453704	1.363327		
H	1.070798	0.209463	1.518676		
H	-0.05468	1.479162	0.985241		
H	-0.49629	0.4315	2.329045		
C	-2.08502	-0.28877	0.071335		
H	-2.22897	0.704042	-0.36925		
H	-2.45202	-1.04155	-0.62492		
H	-2.69013	-0.32247	0.984079		
C	0.07161	-1.5377	-0.14919		
N	0.670672	-2.41677	-0.61909		
AI-ANION					
Atom	X	Y	Z	Electronic Energy (EE)	-210.823
C	-0.55953	-0.37131	0.207539	Zero-point Energy Correction	0.088174
C	0.030525	0.48134	1.310669	Thermal Correction to Energy	0.094337
H	1.11269	0.348833	1.396409	Thermal Correction to Enthalpy	0.095281
H	-0.14888	1.548605	1.129749	Thermal Correction to Free Energy	0.058968
H	-0.40374	0.260764	2.301923		
C	-2.0588	-0.25692	0.031044		
H	-2.35408	0.7697	-0.21954		
H	-2.42711	-0.90093	-0.77246		
H	-2.61951	-0.52353	0.944467		
C	0.038056	-1.57458	-0.07333		
N	0.570705	-2.59439	-0.35321		
AI-RADICAL					
Atom	X	Y	Z	Electronic Energy (EE)	-210.699
C	-0.64658	-0.48303	0.414009	Zero-point Energy Correction	0.08865
C	0.020921	0.453704	1.363327	Thermal Correction to Energy	0.095098
H	1.070798	0.209463	1.518676	Thermal Correction to Enthalpy	0.096042
H	-0.05468	1.479162	0.985241	Thermal Correction to Free Energy	0.057982
H	-0.49629	0.4315	2.329045		
C	-2.08502	-0.28877	0.071335		

H	-2.22897	0.704042	-0.36925
H	-2.45202	-1.04155	-0.62492
H	-2.69013	-0.32247	0.984079
C	0.07161	-1.5377	-0.14919
N	0.670672	-2.41677	-0.61909

BF-ANION

Atom	X	Y	Z		
O	0.777753	-2.51224	0.134314	Electronic Energy (EE)	-1082.47
O	-1.26885	-3.4318	0.413532	Zero-point Energy Correction	0.470578
C	3.725915	-2.03689	-0.09247	Thermal Correction to Energy	0.49658
C	2.642334	-0.95994	-0.19386	Thermal Correction to Enthalpy	0.497525
C	2.293765	2.875306	-0.66429	Thermal Correction to Free Energy	0.415831
C	0.252437	-0.31911	-0.17454		
C	1.949727	1.392068	-0.46612		
C	1.295294	-1.26895	-0.08816		
C	-0.97862	-1.0454	0.010007		
C	2.938091	0.404166	-0.39344		
C	0.601812	1.024479	-0.35229		
C	5.133201	-1.45415	-0.25482		
C	3.516587	-3.08664	-1.19668		
C	3.649607	-2.71757	1.284586		
C	-0.63095	-2.40154	0.213022		
C	1.780908	3.686478	0.537113		
C	1.617994	3.395293	-1.94393		
C	3.800789	3.117122	-0.79194		
C	-2.34871	-0.5511	-0.01279		
C	-2.69746	0.638936	-0.68235		
C	-3.39419	-1.24398	0.625796		
C	-3.99984	1.135392	-0.70805		
C	-5.02736	0.433764	-0.054		
C	-4.69549	-0.75485	0.598296		
C	-4.30831	2.413822	-1.44309		
C	-6.44261	0.947639	-0.06505		
H	3.974432	0.692437	-0.48868		
H	-0.16303	1.789943	-0.38652		
H	5.864285	-2.26355	-0.17747		
H	5.265021	-0.97578	-1.22932		
H	5.361909	-0.72107	0.523653		
H	3.581478	-2.62259	-2.18545		
H	4.292695	-3.8556	-1.13094		
H	2.543713	-3.57182	-1.1063		
H	4.426413	-3.48457	1.364082		
H	3.810323	-1.98758	2.083655		

H	2.68009	-3.19259	1.44095		
H	2.024213	4.745797	0.407926		
H	0.697152	3.600661	0.646639		
H	2.245642	3.340078	1.465111		
H	0.530232	3.305659	-1.88963		
H	1.86187	4.451377	-2.09613		
H	1.962608	2.835834	-2.81881		
H	3.982975	4.185826	-0.93479		
H	4.339419	2.803681	0.106671		
H	4.2248	2.587955	-1.64996		
H	-1.93693	1.183977	-1.22999		
H	-3.1748	-2.16931	1.143534		
H	-5.4793	-1.31082	1.105634		
H	-3.40946	2.827931	-1.90251		
H	-5.05074	2.24876	-2.2299		
H	-4.72465	3.166848	-0.7668		
H	-6.51084	1.944354	0.382409		
H	-6.8307	1.031069	-1.08525		
H	-7.10098	0.280131	0.493583		

BF-RADICAL-V

Atom	X	Y	Z	Electronic Energy (EE)	-1082.32
O	0.971789	-2.41418	-0.07031		
O	-0.94858	-3.60669	-0.02323		
C	3.840952	-1.5573	-0.12236		
C	2.634437	-0.61714	-0.05393		
C	1.822307	3.169287	0.181237		
C	0.184061	-0.28034	0.015845		
C	1.661054	1.644888	0.092737		
C	1.334704	-1.09859	-0.04504		
C	-0.95	-1.16962	0.028531		
C	2.762342	0.785591	0.009844		
C	0.367568	1.104694	0.098797		
C	5.167334	-0.79117	-0.13475		
C	3.76762	-2.40009	-1.40665		
C	3.840796	-2.48695	1.102867		
C	-0.4403	-2.48862	-0.01227		
C	1.207505	3.675299	1.496669		
C	1.095138	3.834515	-0.99911		
C	3.289157	3.608345	0.142896		
C	-2.37008	-0.84645	0.058657		
C	-2.85796	0.397415	-0.38955		
C	-3.32655	-1.76571	0.528809		
C	-4.21109	0.731294	-0.35997		
C	-5.14875	-0.19724	0.124262		

C	-4.67771	-1.4385	0.555303
C	-4.66945	2.078367	-0.85508
C	-6.61613	0.137458	0.168275
H	3.756188	1.207868	-0.00136
H	-0.4849	1.766509	0.184751
H	5.99143	-1.50789	-0.18739
H	5.245085	-0.12726	-1.00022
H	5.300411	-0.19417	0.771684
H	3.780773	-1.75545	-2.29067
H	4.631522	-3.06984	-1.46198
H	2.860859	-3.00593	-1.43474
H	4.705077	-3.15728	1.061438
H	3.906655	-1.90489	2.027043
H	2.93576	-3.09479	1.141571
H	1.320216	4.761427	1.571933
H	0.141775	3.44215	1.557902
H	1.705952	3.219792	2.357608
H	0.026204	3.607464	-0.99295
H	1.208938	4.921848	-0.94704
H	1.510387	3.493069	-1.95208
H	3.340137	4.698896	0.204742
H	3.857079	3.199258	0.983121
H	3.779411	3.302749	-0.78569
H	-2.16673	1.121611	-0.80565
H	-2.99857	-2.73852	0.873024
H	-5.39047	-2.16862	0.929129
H	-3.82542	2.674895	-1.20474
H	-5.38177	1.975697	-1.67944
H	-5.17844	2.637072	-0.06354
H	-6.80814	1.017928	0.789736
H	-7.00585	0.362829	-0.82958
H	-7.19105	-0.69606	0.575462

BF-RADICAL

Atom	X	Y	Z		
O	0.743655	-2.53236	0.102526	Electronic Energy (EE)	-1082.32
O	-1.31758	-3.39267	0.349764	Zero-point Energy Correction	0.47164
C	3.697484	-2.0684	-0.15199	Thermal Correction to Energy	0.497508
C	2.6175	-0.98807	-0.21811	Thermal Correction to Enthalpy	0.498452
C	2.299154	2.869917	-0.60045	Thermal Correction to Free Energy	0.416468
C	0.245587	-0.3127	-0.162		
C	1.927185	1.391525	-0.43424		
C	1.268025	-1.28317	-0.10198		
C	-0.98447	-1.00317	0.014192		

C	2.901927	0.375373	-0.39441
C	0.588681	1.040686	-0.30959
C	5.099946	-1.47805	-0.3212
C	3.468924	-3.09221	-1.27644
C	3.634598	-2.77288	1.213607
C	-0.63541	-2.41251	0.188461
C	1.816892	3.653661	0.630942
C	1.611251	3.427899	-1.85698
C	3.808708	3.080605	-0.74508
C	-2.34179	-0.50653	0.002154
C	-2.684	0.634673	-0.74819
C	-3.35314	-1.14785	0.736478
C	-3.97844	1.137132	-0.76885
C	-4.97997	0.494425	-0.01354
C	-4.64431	-0.64152	0.726302
C	-4.31354	2.348405	-1.59544
C	-6.38578	1.022316	-0.01332
H	3.939347	0.659881	-0.49424
H	-0.18293	1.800225	-0.3075
H	5.831649	-2.28822	-0.27049
H	5.21697	-0.98003	-1.28764
H	5.337154	-0.76197	0.470324
H	3.515334	-2.6062	-2.25516
H	4.249524	-3.85751	-1.23846
H	2.501857	-3.58842	-1.18179
H	4.417179	-3.53521	1.268249
H	3.798703	-2.05721	2.024259
H	2.672288	-3.26173	1.372967
H	2.077249	4.710907	0.524649
H	0.733234	3.58363	0.752878
H	2.289332	3.274368	1.541668
H	0.523059	3.355637	-1.78611
H	1.871343	4.482583	-1.98628
H	1.933079	2.883843	-2.74972
H	4.00955	4.148109	-0.86655
H	4.353211	2.736571	0.138688
H	4.208065	2.563022	-1.62181
H	-1.9311	1.115301	-1.36271
H	-3.1196	-2.0244	1.326785
H	-5.41449	-1.13678	1.30869
H	-3.4311	2.720002	-2.11749
H	-5.08232	2.115719	-2.33806
H	-4.70786	3.15318	-0.96812
H	-6.41709	2.051663	0.35676
H	-6.79818	1.036627	-1.0268
H	-7.03289	0.409212	0.614584

CM-ANION-V

Atom	X	Y	Z		
				Electronic Energy (EE)	-424.821
O	-0.20027	0.987037	-1.59759		
C	-1.47593	1.220387	-1.14669		
C	-2.07977	-0.0026	-0.45194		
C	-1.46555	2.450999	-0.22523		
C	-2.26839	1.547643	-2.4414		
C	-3.38455	0.041305	0.049389		
C	-1.34301	-1.17706	-0.30471		
C	-3.93951	-1.06654	0.681446		
C	-1.89789	-2.28741	0.330098		
C	-3.19668	-2.23682	0.824782		
H	-1.03804	3.302318	-0.7578		
H	-2.48166	2.699439	0.085193		
H	-0.86781	2.247036	0.665816		
H	-1.83104	2.417217	-2.93336		
H	-3.29977	1.770819	-2.16266		
H	-2.25764	0.693899	-3.12066		
H	-3.97705	0.944326	-0.05676		
H	-0.33144	-1.22453	-0.68903		
H	-4.95367	-1.01608	1.061789		
H	-1.31059	-3.19276	0.437003		
H	-3.62856	-3.10032	1.317812		

CM-ANION

Atom	X	Y	Z		
				Electronic Energy (EE)	-424.828
O	-0.13449	0.949279	-1.57294	Zero-point Energy Correction	0.175623
C	-1.40346	1.211093	-1.19681	Thermal Correction to Energy	0.184783
C	-2.07092	0.010184	-0.46897	Thermal Correction to Enthalpy	0.185727
C	-1.46958	2.422237	-0.22483	Thermal Correction to Free Energy	0.141714
C	-2.30109	1.547969	-2.42072		
C	-3.38288	0.043141	0.024941		
C	-1.34048	-1.16586	-0.29585		
C	-3.94099	-1.06008	0.666123		
C	-1.89232	-2.27591	0.344852		
C	-3.19647	-2.22851	0.829542		
H	-1.03359	3.291681	-0.72731		
H	-2.4843	2.681253	0.097656		
H	-0.8671	2.206534	0.663392		
H	-1.86944	2.411014	-2.93783		
H	-3.33952	1.782791	-2.16087		
H	-2.29966	0.700993	-3.11441		

H	-3.98239	0.94119	-0.09136
H	-0.32774	-1.17248	-0.68342
H	-4.95869	-1.00985	1.039646
H	-1.30367	-3.17989	0.466452
H	-3.63004	-3.08848	1.328217

CM-RADICAL

Atom	X	Y	Z		
O	-0.20027	0.987037	-1.59759	Electronic Energy (EE)	-424.673
C	-1.47593	1.220387	-1.14669	Zero-point Energy Correction	0.176303
C	-2.07977	-0.0026	-0.45194	Thermal Correction to Energy	0.185758
C	-1.46555	2.450999	-0.22523	Thermal Correction to Enthalpy	0.186702
C	-2.26839	1.547643	-2.4414	Thermal Correction to Free Energy	0.141065
C	-3.38455	0.041305	0.049389		
C	-1.34301	-1.17706	-0.30471		
C	-3.93951	-1.06654	0.681446		
C	-1.89789	-2.28741	0.330098		
C	-3.19668	-2.23682	0.824782		
H	-1.03804	3.302318	-0.7578		
H	-2.48166	2.699439	0.085193		
H	-0.86781	2.247036	0.665816		
H	-1.83104	2.417217	-2.93336		
H	-3.29977	1.770819	-2.16266		
H	-2.25764	0.693899	-3.12066		
H	-3.97705	0.944326	-0.05676		
H	-0.33144	-1.22453	-0.68903		
H	-4.95367	-1.01608	1.061789		
H	-1.31059	-3.19276	0.437003		
H	-3.62856	-3.10032	1.317812		

DPPH-ANION-V

Atom	X	Y	Z		
O	0.658816	0.7152	-2.21623	Electronic Energy (EE)	-1417.88
O	-1.86889	-3.67604	1.071789		
O	-0.56816	2.436828	-1.82416		
O	-0.51376	-2.41101	2.161645		
O	-5.66297	-0.60938	-0.05391		
O	-5.19277	1.182738	-1.14361		
N	1.560751	-0.2045	0.147028		
N	0.588688	-1.08818	0.026825		
N	-0.28682	1.264883	-1.68924		
N	-1.32209	-2.61445	1.283609		
N	-4.87568	0.174488	-0.54529		

C	-0.69232	-0.66928	-0.13008
C	2.860161	-0.64888	-0.2141
C	1.362625	1.075963	0.761571
C	-1.18461	0.438772	-0.87259
C	-1.71175	-1.4787	0.431664
C	-3.45113	-0.11603	-0.40443
C	-2.52853	0.731511	-0.99162
C	-3.05767	-1.24857	0.297342
C	3.981017	-0.19549	0.48462
C	0.550404	1.166001	1.891193
C	2.988246	-1.55663	-1.26657
C	1.97255	2.20407	0.216055
C	5.237204	-0.6612	0.119596
C	0.339882	2.409843	2.472737
C	4.251581	-2.01408	-1.61665
C	1.756884	3.439968	0.811755
C	5.377674	-1.56891	-0.92797
C	0.939561	3.546083	1.934864
H	-2.84879	1.588183	-1.57067
H	-3.78278	-1.91042	0.753714
H	3.87137	0.495959	1.310991
H	0.105767	0.27116	2.313321
H	2.105858	-1.8812	-1.80331
H	2.591251	2.113695	-0.66954
H	6.109067	-0.31903	0.664431
H	-0.28533	2.487058	3.354194
H	4.356005	-2.71439	-2.43698
H	2.221045	4.323011	0.389142
H	6.361646	-1.92693	-1.20703
H	0.772158	4.513931	2.392684

DPPH-ANION

Atom	X	Y	Z		
O	-0.57745	0.615391	2.270085	Zero-point Energy Correction	0.290831
O	1.861282	-3.7169	-1.32386	Thermal Correction to Energy	0.314091
O	0.7703	2.287986	2.104216	Thermal Correction to Enthalpy	0.315035
O	0.336261	-3.58812	0.183232	Thermal Correction to Free Energy	0.235099
O	5.534527	-0.6222	-0.51456		
O	5.127419	1.322183	0.316536		
N	-1.57184	-0.01193	-0.1777		
N	-0.59105	-0.97086	-0.28186		
N	0.403669	1.162105	1.789393		
N	1.241611	-3.10481	-0.46769		
N	4.763628	0.207937	-0.04423		

Electronic Energy (EE): -1417.9

C	0.605167	-0.75088	0.165018
C	-2.87454	-0.53194	-0.1128
C	-1.31449	1.335216	-0.54766
C	1.186549	0.409651	0.844926
C	1.634411	-1.7323	-0.18721
C	3.384747	-0.13412	0.073744
C	2.5134	0.738213	0.724571
C	2.950703	-1.42086	-0.30828
C	-3.97973	0.145729	-0.64787
C	-0.38862	1.63351	-1.54923
C	-3.07156	-1.78145	0.493527
C	-1.97341	2.374159	0.115936
C	-5.24853	-0.41448	-0.55975
C	-0.13204	2.959838	-1.88464
C	-4.3457	-2.33227	0.563505
C	-1.72589	3.692939	-0.23825
C	-5.44566	-1.65404	0.044735
C	-0.8005	3.994026	-1.2372
H	2.888582	1.640135	1.190303
H	3.660966	-2.16115	-0.65222
H	-3.85154	1.099627	-1.14308
H	0.131482	0.831311	-2.06061
H	-2.21851	-2.30906	0.901848
H	-2.66738	2.13937	0.914697
H	-6.08993	0.123253	-0.9828
H	0.590877	3.180403	-2.66189
H	-4.47712	-3.29915	1.036948
H	-2.24333	4.490524	0.282763
H	-6.43829	-2.08394	0.106965
H	-0.59911	5.025431	-1.5022

DPPH-RADICAL

Atom	X	Y	Z		
O	0.675801	0.733717	-2.21342	Electronic Energy (EE)	-1417.73
O	-1.89623	-3.67033	1.022687	Zero-point Energy Correction	0.292195
O	-0.54477	2.45857	-1.81557	Thermal Correction to Energy	0.315396
O	-0.54174	-2.42117	2.131484	Thermal Correction to Enthalpy	0.31634
O	-5.66685	-0.57492	-0.10314	Thermal Correction to Free Energy	0.236423
O	-5.1802	1.223358	-1.17532		
N	1.557394	-0.20955	0.148226		
N	0.581566	-1.08714	0.014591		
N	-0.27041	1.284125	-1.68822		
N	-1.34533	-2.61335	1.246583		
N	-4.87229	0.208712	-0.58305		

C	-0.69619	-0.66028	-0.14731
C	2.856825	-0.65784	-0.20796
C	1.361884	1.066985	0.771714
C	-1.17782	0.456237	-0.88411
C	-1.72348	-1.4688	0.40125
C	-3.45023	-0.09039	-0.43523
C	-2.5194	0.756963	-1.00957
C	-3.06728	-1.23055	0.25999
C	3.975403	-0.21594	0.501681
C	0.542734	1.152256	1.896693
C	2.98711	-1.55782	-1.26682
C	1.981232	2.196199	0.239261
C	5.231527	-0.6853	0.141145
C	0.334855	2.3925	2.486809
C	4.250326	-2.01907	-1.61229
C	1.768075	3.428413	0.843435
C	5.374164	-1.58533	-0.91272
C	0.943949	3.529839	1.961997
H	-2.8314	1.619901	-1.58381
H	-3.7988	-1.89221	0.706292
H	3.863918	0.46946	1.332838
H	0.090691	0.256416	2.308715
H	2.106596	-1.87345	-1.8119
H	2.605263	2.109657	-0.64297
H	6.101554	-0.35207	0.694391
H	-0.29573	2.46595	3.364755
H	4.356502	-2.71334	-2.43752
H	2.239584	4.312357	0.430961
H	6.358077	-1.94626	-1.18822
H	0.778569	4.494864	2.426463

H1-DPPH-CATION-RADICAL

Atom	X	Y	Z		
O	-0.02764	1.148936	-2.48315	Electronic Energy (EE)	-1418.1
O	-0.77595	-3.61205	-0.08924	Zero-point Energy Correction	0.304528
O	-1.14989	2.744376	-1.57616	Thermal Correction to Energy	0.327899
O	-0.82301	-2.86283	1.932579	Thermal Correction to Enthalpy	0.328843
O	-5.6289	-0.82573	0.826548	Thermal Correction to Free Energy	0.24911
O	-5.45569	1.130861	-0.05298		
N	1.513411	-0.0284	-0.59251		
N	0.460674	-0.9346	-0.25504		
N	-0.83166	1.590107	-1.67837		
N	-1.03149	-2.78143	0.746522		

N	-5.00334	0.066205	0.30123
C	-0.8113	-0.56938	-0.34644
C	2.815022	-0.7172	-0.36217
C	1.452655	1.285861	0.129409
C	-1.49647	0.606396	-0.81437
C	-1.67427	-1.53864	0.27013
C	-3.5619	-0.16582	0.074958
C	-2.82924	0.817898	-0.59091
C	-3.00999	-1.36352	0.507402
C	3.031019	-1.35515	0.848153
C	0.937071	1.337971	1.414223
C	3.762226	-0.64657	-1.36656
C	1.957298	2.392299	-0.53611
C	4.267111	-1.9564	1.049752
C	0.913595	2.575114	2.051246
C	4.996251	-1.25201	-1.14415
C	1.922789	3.617282	0.119132
C	5.245861	-1.90355	0.058481
C	1.399743	3.708019	1.406677
H	-3.31214	1.716042	-0.95665
H	-3.60361	-2.11971	1.007836
H	2.259608	-1.38781	1.609226
H	0.56671	0.450307	1.914605
H	3.543662	-0.13454	-2.29772
H	2.358147	2.304596	-1.54051
H	4.464081	-2.46698	1.9845
H	0.514118	2.644999	3.055421
H	5.754882	-1.21438	-1.91625
H	2.300974	4.498978	-0.38327
H	6.206547	-2.37582	0.226255
H	1.371259	4.667134	1.909621
H	1.448399	0.177087	-1.6077

H1-DPPH-V

Atom	X	Y	Z	Electronic Energy (EE)	-1418.33
O	-0.02764	1.148936	-2.48315		
O	-0.77595	-3.61205	-0.08924		
O	-1.14989	2.744376	-1.57616		
O	-0.82301	-2.86283	1.932579		
O	-5.6289	-0.82573	0.826548		
O	-5.45569	1.130861	-0.05298		
N	1.513411	-0.0284	-0.59251		
N	0.460674	-0.9346	-0.25504		
N	-0.83166	1.590107	-1.67837		
N	-1.03149	-2.78143	0.746522		

N	-5.00334	0.066205	0.30123
C	-0.8113	-0.56938	-0.34644
C	2.815022	-0.7172	-0.36217
C	1.452655	1.285861	0.129409
C	-1.49647	0.606396	-0.81437
C	-1.67427	-1.53864	0.27013
C	-3.5619	-0.16582	0.074958
C	-2.82924	0.817898	-0.59091
C	-3.00999	-1.36352	0.507402
C	3.031019	-1.35515	0.848153
C	0.937071	1.337971	1.414223
C	3.762226	-0.64657	-1.36656
C	1.957298	2.392299	-0.53611
C	4.267111	-1.9564	1.049752
C	0.913595	2.575114	2.051246
C	4.996251	-1.25201	-1.14415
C	1.922789	3.617282	0.119132
C	5.245861	-1.90355	0.058481
C	1.399743	3.708019	1.406677
H	-3.31214	1.716042	-0.95665
H	-3.60361	-2.11971	1.007836
H	2.259608	-1.38781	1.609226
H	0.56671	0.450307	1.914605
H	3.543662	-0.13454	-2.29772
H	2.358147	2.304596	-1.54051
H	4.464081	-2.46698	1.9845
H	0.514118	2.644999	3.055421
H	5.754882	-1.21438	-1.91625
H	2.300974	4.498978	-0.38327
H	6.206547	-2.37582	0.226255
H	1.371259	4.667134	1.909621
H	1.448399	0.177087	-1.6077

H1-DPPH

Atom	X	Y	Z	Electronic Energy (EE)	-1418.34
O	0.117032	0.779175	-2.61404	Zero-point Energy Correction	0.305114
O	-0.6682	-3.5808	-0.22446	Thermal Correction to Energy	0.328413
O	-1.17861	2.42676	-2.14984	Thermal Correction to Enthalpy	0.329357
O	-1.5439	-3.32576	1.720673	Thermal Correction to Free Energy	0.24884
O	-5.508	-0.41986	1.113693		
O	-5.21624	1.494394	0.17925		
N	1.512837	-0.07498	-0.56592		
N	0.498714	-1.02878	-0.2291		
N	-0.77633	1.301199	-1.93881		

N	-1.26152	-2.9297	0.606252
N	-4.82489	0.388234	0.508291
C	-0.75526	-0.67845	-0.37992
C	2.840313	-0.69344	-0.33784
C	1.402279	1.228898	0.155209
C	-1.39909	0.503373	-0.91957
C	-1.71395	-1.58528	0.230555
C	-3.46988	0.012069	0.172792
C	-2.69946	0.86021	-0.58859
C	-2.9885	-1.25261	0.55454
C	3.098116	-1.32712	0.867663
C	0.947907	1.25076	1.465005
C	3.786237	-0.57689	-1.3407
C	1.795017	2.372357	-0.52151
C	4.363195	-1.8671	1.06501
C	0.880168	2.478859	2.113254
C	5.04984	-1.12228	-1.12708
C	1.72038	3.592921	0.142617
C	5.336723	-1.76449	0.072139
C	1.262771	3.645677	1.455395
H	-3.11179	1.784112	-0.97221
H	-3.62244	-1.95363	1.082845
H	2.329164	-1.40498	1.626679
H	0.646953	0.337871	1.964777
H	3.540988	-0.07567	-2.27117
H	2.148192	2.315635	-1.54634
H	4.587725	-2.37119	1.997329
H	0.524853	2.520724	3.135742
H	5.802259	-1.04559	-1.90263
H	2.016442	4.498784	-0.37233
H	6.319931	-2.18993	0.235243
H	1.201656	4.598324	1.968087
H	1.444992	0.146123	-1.57734

H2-DPPH-CATION-RADICAL

Atom	X	Y	Z		
O	0.814075	0.501448	-2.16341	Zero-point Energy Correction	0.304793
O	-0.56863	-3.20201	1.237842	Thermal Correction to Energy	0.328097
O	-0.28088	2.32084	-1.82372	Thermal Correction to Enthalpy	0.329041
O	-2.46311	-2.83338	2.172195	Thermal Correction to Free Energy	0.249762
O	-5.67185	-0.23201	-0.20316		
O	-5.00594	1.487863	-1.30805		
N	1.599927	-0.22722	0.261421		

Electronic Energy (EE): -1418.16

N	0.561475	-1.08743	0.171759
N	-0.10017	1.134774	-1.67871
N	-1.60216	-2.5687	1.369653
N	-4.8085	0.47238	-0.67782
C	-0.73966	-0.71701	-0.09361
C	2.833293	-0.67991	-0.20693
C	1.404015	1.023981	0.890173
C	-1.09931	0.389113	-0.89178
C	-1.8241	-1.4162	0.485643
C	-3.41038	0.065004	-0.47133
C	-2.40458	0.799424	-1.06849
C	-3.14202	-1.04573	0.301131
C	4.014703	-0.22621	0.407921
C	0.542865	1.112505	1.990792
C	2.867491	-1.6075	-1.26257
C	2.061015	2.147021	0.374147
C	5.227398	-0.69948	-0.05479
C	0.354464	2.34839	2.584285
C	4.093065	-2.06716	-1.70512
C	1.857266	3.373908	0.984235
C	5.271748	-1.61531	-1.10813
C	1.007033	3.476366	2.083847
H	-2.62615	1.655922	-1.69288
H	-3.93737	-1.61298	0.767285
H	3.968135	0.449581	1.252697
H	0.064584	0.224043	2.387876
H	1.948128	-1.92209	-1.73996
H	2.691622	2.055769	-0.50309
H	6.143822	-0.36932	0.418077
H	-0.29337	2.431691	3.448063
H	4.134228	-2.76842	-2.5293
H	2.350932	4.253674	0.590803
H	6.228065	-1.98057	-1.46295
H	0.849435	4.439811	2.553963
H	0.764219	-2.05219	0.428082

H2-DPPH-V

Atom	X	Y	Z	Electronic Energy (EE)	-1418.35
O	0.814075	0.501448	-2.16341		
O	-0.56863	-3.20201	1.237842		
O	-0.28088	2.32084	-1.82372		
O	-2.46311	-2.83338	2.172195		
O	-5.67185	-0.23201	-0.20316		
O	-5.00594	1.487863	-1.30805		
N	1.599927	-0.22722	0.261421		

N	0.561475	-1.08743	0.171759
N	-0.10017	1.134774	-1.67871
N	-1.60216	-2.5687	1.369653
N	-4.8085	0.47238	-0.67782
C	-0.73966	-0.71701	-0.09361
C	2.833293	-0.67991	-0.20693
C	1.404015	1.023981	0.890173
C	-1.09931	0.389113	-0.89178
C	-1.8241	-1.4162	0.485643
C	-3.41038	0.065004	-0.47133
C	-2.40458	0.799424	-1.06849
C	-3.14202	-1.04573	0.301131
C	4.014703	-0.22621	0.407921
C	0.542865	1.112505	1.990792
C	2.867491	-1.6075	-1.26257
C	2.061015	2.147021	0.374147
C	5.227398	-0.69948	-0.05479
C	0.354464	2.34839	2.584285
C	4.093065	-2.06716	-1.70512
C	1.857266	3.373908	0.984235
C	5.271748	-1.61531	-1.10813
C	1.007033	3.476366	2.083847
H	-2.62615	1.655922	-1.69288
H	-3.93737	-1.61298	0.767285
H	3.968135	0.449581	1.252697
H	0.064584	0.224043	2.387876
H	1.948128	-1.92209	-1.73996
H	2.691622	2.055769	-0.50309
H	6.143822	-0.36932	0.418077
H	-0.29337	2.431691	3.448063
H	4.134228	-2.76842	-2.5293
H	2.350932	4.253674	0.590803
H	6.228065	-1.98057	-1.46295
H	0.849435	4.439811	2.553963
H	0.764219	-2.05219	0.428082

H2-DPPH

Atom	X	Y	Z		
				Electronic Energy (EE)	-1418.36
O	0.63311	-0.13735	-2.60432	Zero-point Energy Correction	0.305172
O	-0.67379	-2.90513	1.715053	Thermal Correction to Energy	0.328427
O	0.144204	1.887156	-2.06002	Thermal Correction to Enthalpy	0.329371
O	-2.70447	-2.56553	2.316317	Thermal Correction to Free Energy	0.250242
O	-5.66623	-0.17103	-0.50745		
O	-4.88964	1.289534	-1.87957		

N	1.48785	-0.02514	0.108925
N	0.527617	-1.00619	0.30782
N	0.015136	0.687258	-1.96831
N	-1.74971	-2.33609	1.607777
N	-4.751	0.400388	-1.06393
C	-0.74816	-0.76006	-0.0633
C	2.740814	-0.52658	-0.3436
C	1.400694	1.103957	0.991002
C	-1.05243	0.177252	-1.08418
C	-1.89048	-1.33607	0.556559
C	-3.38798	-0.00339	-0.72799
C	-2.32185	0.576007	-1.40838
C	-3.18601	-0.96525	0.235481
C	3.94634	0.07642	0.026633
C	0.834082	0.993887	2.25909
C	2.761371	-1.65051	-1.17729
C	1.850838	2.341248	0.527644
C	5.149548	-0.4288	-0.45622
C	0.732443	2.124256	3.066424
C	3.971732	-2.14697	-1.64466
C	1.761151	3.45823	1.347948
C	5.173839	-1.5376	-1.29539
C	1.199342	3.35527	2.619342
H	-2.48682	1.294952	-2.20161
H	-4.01926	-1.4233	0.751491
H	3.95893	0.92864	0.692626
H	0.471586	0.039611	2.62359
H	1.833322	-2.13109	-1.45725
H	2.267738	2.419779	-0.46987
H	6.075503	0.050496	-0.15926
H	0.29021	2.033539	4.051806
H	3.969353	-3.01765	-2.29063
H	2.117399	4.415418	0.98515
H	6.115126	-1.92634	-1.66515
H	1.121225	4.230874	3.252996
H	0.709769	-1.69946	1.027515

HOO-ANION-V

Atom	X	Y	Z	Electronic Energy (EE)	-151.022
H	-0.8796	-0.88239	0		
O	0.053405	0.706561	0		
O	0.057281	-0.59788	0		

HOO-ANION

Atom	X	Y	Z		
H	-0.87955	-0.89034	0	Zero-point Energy Correction	0.013686
O	0.049412	0.786081	0	Thermal Correction to Energy	0.016582
O	0.058498	-0.68308	0	Thermal Correction to Enthalpy	0.017527
				Thermal Correction to Free Energy	-0.00803

Electronic Energy (EE): -151.039

HOO-RADICAL

Atom	X	Y	Z	Electronic Energy (EE)	-150.907
H	-0.8796	-0.88239	0	Zero-point Energy Correction	0.014438
O	0.053405	0.706561	0	Thermal Correction to Energy	0.017288
O	0.057281	-0.59788	0	Thermal Correction to Enthalpy	0.018233
				Thermal Correction to Free Energy	-0.0077

TB-ANION-V

Atom	X	Y	Z	Electronic Energy (EE)	-233.104
O	0.513287	1.359813	0		
C	0.062559	0.055909	0		
C	-1.47837	0.215159	0		
C	0.513287	-0.6737	1.266932		
C	0.513287	-0.6737	-1.26693		
H	-1.81038	0.749335	-0.89149		
H	-1.81038	0.749335	0.891494		
H	-1.91253	-0.78716	0		
H	0.167841	-0.14031	2.155077		
H	0.109325	-1.68865	1.283682		
H	1.604055	-0.73703	1.296445		
H	0.167841	-0.14031	-2.15508		
H	0.109325	-1.68865	-1.28368		
H	1.604055	-0.73703	-1.29645		

TB-ANION

Atom	X	Y	Z	Electronic Energy (EE)	-233.111
O	0.52383	1.40059	0	Zero-point Energy Correction	0.121883
C	0.048041	0.129301	0	Thermal Correction to Energy	0.128066
C	-1.50223	0.100702	0	Thermal Correction to Enthalpy	0.12901
C	0.52383	-0.65797	1.248322	Thermal Correction to Free Energy	0.093385
C	0.52383	-0.65797	-1.24832		
H	-1.87478	0.627893	-0.88495		
H	-1.87478	0.627893	0.884951		
H	-1.92173	-0.91338	0		

H	0.18595	-0.14233	2.153812
H	0.155147	-1.69111	1.281296
H	1.61881	-0.6823	1.265863
H	0.18595	-0.14233	-2.15381
H	0.155147	-1.69111	-1.2813
H	1.61881	-0.6823	-1.26586

TB-RADICAL

Atom	X	Y	Z		
O	0.513287	1.359813	0	Electronic Energy (EE)	-232.963
C	0.062559	0.055909	0	Zero-point Energy Correction	0.122817
C	-1.47837	0.215159	0	Thermal Correction to Energy	0.129215
C	0.513287	-0.6737	1.266932	Thermal Correction to Enthalpy	0.13016
C	0.513287	-0.6737	-1.26693	Thermal Correction to Free Energy	0.093424
H	-1.81038	0.749335	-0.89149		
H	-1.81038	0.749335	0.891494		
H	-1.91253	-0.78716	0		
H	0.167841	-0.14031	2.155077		
H	0.109325	-1.68865	1.283682		
H	1.604055	-0.73703	1.296445		
H	0.167841	-0.14031	-2.15508		
H	0.109325	-1.68865	-1.28368		
H	1.604055	-0.73703	-1.29645		

References

1. M. G. Evans and M. Polanyi, *Trans. Faraday Soc.*, 1935, **31**, 875-894.
2. H. Eyring, *J. Chem. Phys.*, 1935, **3**, 107-115.
3. D. G. Truhlar, W. L. Hase and J. T. Hynes, *J. Phys. Chem.*, 1983, **87**, 2664-2682.
4. T. Furuncuoglu, I. Ugur, I. Degirmenci and V. Aviyente, *Macromolecules*, 2010, **43**, 1823-1835.
5. E. Vélez, J. Quijano, R. Notario, E. Pabón, J. Murillo, J. Leal, E. Zapata and G. Alarcón, *J. Phys. Org. Chem.*, 2009, **22**, 971-977.
6. E. Pollak and P. Pechukas, *J. Am. Chem. Soc.*, 1978, **100**, 2984-2991.
7. A. Fernández-Ramos, B. A. Ellingson, R. Meana-Pañeda, J. M. Marques and D. G. Truhlar, *Theor. Chem. Acc.*, 2007, **118**, 813-826.
8. C. Eckart, *Phy. Rev.*, 1930, **35**, 1303.
9. R. A. Marcus, *Annu. Rev. Phys. Chem.*, 1964, **15**, 155-196.
10. R. A. Marcus, *Rev. Mod. Phys.*, 1993, **65**, 599.
11. Y. Lu, A. Wang, P. Shi and H. Zhang, *PloS one*, 2017, **12**, e0169773.

12. Y. Lu, A. Wang, P. Shi, H. Zhang and Z. Li, *PloS one*, 2015, **10**, e0133259.

13. S. F. Nelsen, S. C. Blackstock and Y. Kim, *J. Am. Chem. Soc.*, 1987, **109**, 677-682.

14. S. F. Nelsen, M. N. Weaver, Y. Luo, J. R. Pladziewicz, L. K. Ausman, T. L. Jentzsch and J. J. O'Konek, *J. Phys. Chem. A*, 2006, **110**, 11665-11676.

15. A. Galano and J. R. Alvarez-Idaboy, *J. Comput. Chem.*, 2013, **34**, 2430-2445.

16. F. C. Collins and G. E. Kimball, *J. Colloid Sci.* , 1949, **4**, 425-437.

17. M. Von Smoluchowski, *Z. Phys. Chem*, 1917, **92**, 129-168.

18. D. G. Truhlar, *J. Chem. Educ.*, 1985, **62**, 104.

19. A. Einstein, *Ann. Phys.*, 1905, **17**, 549-560.

20. G. G. Stokes, *Mathematical and Physical Papers*, University Press, Cambridge, 1905.

21. F. G. Bordwell, *Accounts of Chemical Research*, 1988, **21**, 456-463.

22. W. S. Matthews, J. E. Bares, J. E. Bartmess, F. Bordwell, F. J. Cornforth, G. E. Drucker, Z. Margolin, R. J. McCallum, G. J. McCollum and N. R. Vanier, *Journal of the American Chemical Society*, 1975, **97**, 7006-7014.
